# Predicting the effectiveness of omalizumab in patients with refractory chronic rhinosinusitis with nasal polyps comorbid with asthma based on inflammatory biomarkers^☆^

**DOI:** 10.1016/j.waojou.2024.101009

**Published:** 2024-12-12

**Authors:** Yutong Sima, Ming Zheng, Yan Zhao, Siqi Ge, Chengyao Liu, Ping Wang, Xiangdong Wang, Luo Zhang

**Affiliations:** a Department of Otolaryngology Head and Neck Surgery, Beijing Tongren Hospital, Capital Medical University, Beijing 100730, China; b Beijing Laboratory of Allergic Diseases, Beijing Municipal Education Commission and Beijing Key Laboratory of Nasal Diseases, Beijing Institute of Otolaryngology, Beijing 100005, China; c Department of Neuroepidemiology, Beijing Neurosurgical Institute, Capital Medical University, Beijing 100070, China; d Department of Allergy, Beijing Tongren Hospital, Capital Medical University, Beijing 100730, China; e Research Unit of Diagnosis and Treatment of Chronic Nasal Diseases, Chinese Academy of Medical Sciences, Beijing 100005, China

**Keywords:** Chronic rhinosinusitis with nasal polyps, Omalizumab, Endotype, Prediction

## Abstract

**Background:**

The treatment of refractory chronic rhinosinusitis with nasal polyps (CRSwNP) with omalizumab has been well studied based on clinical evaluation. Nevertheless, ideal quantitative or qualitative biomarkers for predicting a different response to biologics urgently need to be explored. We aim to identify potential biomarkers for predicting a good or poor response in patients with refractory CRSwNP.

**Methodology:**

Patients received an endoscopic and radiological evaluation, a visual analogue scale (VAS) assessment, and a 22-item sinonasal outcome test (SNOT-22). Forty-eight biomarkers involving type 1 (T1), type 2 (T2), and type 3 (T3) inflammatory factors, chemokines, and remodeling factors were detected in nasal secretion and serum samples at baseline and after 24 weeks of omalizumab treatment.

**Results:**

Eighteen patients with CRSwNP and 16 patients as control were enrolled. Patients with CRSwNP who received oamlizumab treatment with the SNOT-22 and VAS scores improved by 8.9 and 2 points in 72.22% and 50%, respectively. The nasal polyp score (NPS) and Lund-Mackay score were significantly improved in 55.56% of patients. The concentrations of T2 inflammatory biomarker, granulocyte-macrophage colony-stimulating factor (GM-CSF), T3 inflammatory biomarkers, granulocyte colony-stimulating factor (G-CSF), chemokine (C-X-C motif) ligand (CXCL)-1, and chemokine (C–C motif) ligand-20 (CCL-20), T1 inflammatory biomarker, IP-10 (CXCL-10), and granzyme B in nasal secretion and serum periostin were significantly decreased. Serum CCL-3 (AUC = 0.836) and CCL-4 (AUC = 0.909) levels predicted the improvement of SNOT-22 score, respectively. Serum IL-8 (AUC = 0.883) predicted poor improvement in nasal congestion score. Nasal secretion CXCL-1 (AUC = 0.812), GM-CSF (AUC = 0.813), IgE (AUC = 0.900) and IP-10 (AUC = 0.800) effectively predicted none or less improvement in nasal polyp score.

**Conclusions:**

Omalizumab remarkably affects inflammatory mediators in different pathways. CCL-3 and CCL-4 in serum and IgE, CXCL-1, GM-CSF, and IP-10 in nasal secretion may be considered as preferable biomarkers for predicting favorable or ineffective response to omalizumab therapy in patients with refractory CRSwNP comorbid with asthma, based on various clinical indicators.

## Introduction

Chronic rhinosinusitis (CRS) is a common chronic respiratory disease with a prevalence of approximately 10.9%, 12%, and 2.1% in Europe, the United States, and China, respectively.^1^, ^2^, ^3^ CRS is characterized by nasal obstruction, rhinorrhea, facial pain, and smell reduction lasting 12 weeks, which significantly reduces patient quality of life (QOL) and increases financial burden.**^4^** CRS is divided into chronic rhinosinusitis without nasal polyps (CRSsNP) and chronic rhinosinusitis with nasal polyps (CRSwNP). CRSwNP is associated with a higher mortality risk and prevalence of comorbidities, including asthma and allergic rhinitis, than CRSsNP;^5^ thus, superior therapeutic strategies for CRSwNP are needed. Surgery and medication, including intranasal or systemic corticosteroids, antibiotics, and saline irrigation, are the major treatment methods for CRSwNP. However, the popularization and promotion of endoscopic sinus surgery (ESS) and maximal medical regimens still not reduced the frequent recurrence of CRSwNP, even after standard care based on clinical guidelines.^6^ Hull reported that 38%–69% of CRSwNP patients required revision ESS.^7^ A recent meta-analysis of 45 studies demonstrated that the long-term revision rates of ESS are approximately 14%–24%.^8^ Zhang also showed that the recurrence rates of Chinese CRSwNP patients with asthma are 95.6%–96.1% after functional ESS, radical endoscopic sinus surgery (RESS), and RESS + Draf III during a 5-year follow-up period.^9^ Moreover, CRSwNP patients require repetitive prescription of oral corticosteroids to avoid disease exacerbation and reoperation, which may consequently induce many complications, such as osteoporosis, diabetes, and hypertension.^10^ Approximately 29% and 41.8% of CRSwNP patients in China and Belgium, respectively, suffer from the refractory form, even after receiving maximal medical treatment and revision ESS.^11^^,^^12^ Therefore, appropriate and personalized treatment for CRS is imperative.

CRS displays obvious heterogeneity in pathophysiology, with various endotypes that have overlapping clinical manifestations and distinct prognoses. Tomassen et al introduced that CRS patients could be divided into 3 subgroups with 10 clusters: non-type 2, moderate and severe type 2, in which the latter subgroup showed the highest concentrations of IgE and asthma prevalence.^13^ Meanwhile, the endotypes of CRS based on T helper 1(Th1)/Th2/Th17 immunity have been shown diversity in different regions worldwide.^14^ One recent study also showed non-type 2, mild, moderate, and severe type 2 endotypes in 95 CRSwNP and 33 CRSsNP, in which 48 inflammatory and remodeling biomarkers exhibited different trends in mucosal tissue.^15^ The endotype of CRSwNP cases shows a dominant type 2 inflammation signature in many cohort characterized by a Th2-biased cascade reaction and hyperproduction of IgE and eosinophilia associated with more severely compromised quality of life, higher asthma comorbidity and recurrence after surgeries or oral corticosteroids.^16^ As a result, many novel biologics targeting pivotal markers in the type 2 inflammation pathway, such as IgE, IL-5, and IL-4Rα, originally developed for uncontrolled asthma, are recommended for treating refractory or uncontrolled CRSwNP.^15^ However, the clinical improvement of these biologics for CRSwNP showed heterogeneity in these studies because of the diversity of inflammation within and between the endotypes which could not be reflected by the criteria of type 2 utilized now, which were mainly based on similar biomarkers such as serum IgE level or blood eosinophils combined with clinical disorders. Although both omalizumab (anti-IgE monoclonal antibody [mAb]) and dupilumab (anti-IL-4Rα mAb) have been approved by the US Food and Drug Administration (FDA) and European Medicines Agency (EMA) for the treatment of severe CRSwNP,^15^ omalizumab is more commonly administered to Chinese CRSwNP patients since it was the first approved biological therapy for type 2 (T2) inflammation and has been widely used in China. In addition, several newly published data have shown that omalizumab is an attractive and rational biological therapy for treating refractory CRSwNP.^17^^,^^18^ Nevertheless, the comprehensive immunological responses including type 1 (T1, such as IFN-γ et al.), type 2 (T2, such as IL-5 et al), and type 3 (T3, such as IL-17 et al.) inflammatory biomarkers, chemokines (C–C motif ligand 2, CCL-2 et al.), proinflammatory factors (IL-1α et al.), and even remodeling (epidermal growth factor, EGF et al) profile after biological therapy for CRSwNP have not been evaluated, which can acquire a better understanding the mechanism of biologics and may find ideal quantitative or qualitative biomarkers for predicting the response to treatment. The latest omalizumab study reported a higher score (18.5) on the asthma control test (ACT), a noninvasive assessment, represents a moderate predictive indicator (area under the receiver operating characteristic curve (AUC) = 0.771) for improvement in loss of smell in patients with difficult-to-treat CRSwNP.^19^ In clinic, the biomarkers in serum or nasal secretion of CRSwNP patients are easier to obtaine and measure repeatedly compared to tissue to predict and reflect the efficacy of biologics.

Therefore, the main aim of the present prospective study was to explore reliable and accurate endotype biomarkers for predicting a favorable response to omalizumab. The molecular changes in serum and nasal secretion to reflect the inflammatory pathways after anti-IgE treatment were also assessed.

## Materials and methods

### Ethics statement

Each patient provided written informed consent, and the study was approved by the Ethics Committee and clinical trial registration.

### Subjects

The inclusion criteria were patients older than 18 years with recurrent, refractory CRSwNP and comorbid with asthma. The diagnosis of CRSwNP was based on the European Position Paper on Rhinosinusitis and Nasal Polyps 2012 guidelines (EPOS 2012)^20^ and patients have the positive specific-IgE and the symptoms includes paroxysmal sneezing, rhinorrhea, nasal congestion, and itchy were defined as allergic rhinitis based on the AAIR guildline.^21^ Asthma was diagnosed by pulmonologist based on the Global Initiative for Asthma guidelines (GINA).^22^ Refractory CRSwNP was defined as persistent symptoms of CRSwNP after receiving adequate ESS and maximal medical treatment, including continuous and long-term intranasal corticosteroid treatment comorbid with 2 short courses of antibiotics or oral corticosteroids in the past year. The patients received omalizumab every 4 weeks for 24 consecutive weeks based on the serum total IgE levels and body weight (kilograms) according to the manufacture's instruction. The patients also received mometasone furoate nasal spray (100 μg/nostril) twice daily and inhaled asthma control therapies throughout the study. The exclusion criteria were patients with autoimmune diseases, psychological disorders, those who received oral corticosteroids in the previous 4 weeks, and those who received monoclonal antibody therapy in the previous 6 months. Pregnant patients were also excluded from the study.

Meanwhile, we collected peripheral blood and nasal secretions from patients who required surgical treatment for deviated septum as control subjects. All of them were examined by allergens and sinus CT to confirm the absence of combined with AR or CRS.

### Assessment

Demographic data were collected during the baseline screening. The nasal polyp score (NPS), Lund-MacKay score (LMS), and the Lund-Kennedy score (LKS) (range 0–8, 0–24, 0–20, respectively, with the lower values indicating milder sinus inflammatory degree) analyses were conducted at the baseline and after 24 weeks of omalizumab therapy. The data were scored by 2 independent otolaryngologists blinded to the treatment period. QOL and nasal symptoms were also recorded at the baseline and after 24 weeks of treatment which were measured based on a 22-item sino-nasal outcome test (SNOT-22, range 0–110, with lower values indicating less damage to QOL) and visual analogue scale (VAS), including nasal congestion score (NCS), runny nose score (RNS), sense of smell score (SSS) and facial pressure score (FPS) (range 0–10, with lower scores indicating mild subjective nasal symptoms and higher scores indicating the worst nasal symptoms). ACT score was also assessed at the baseline and after 24 weeks of treatment (range 0–25, with higher scores indicating milder asthma symptoms and better perception of control).

Greater improvements than the accepted minimal clinically important difference (MCID) (8.9 points on the SNOT-22 and higher than 2 points on the VAS) between baseline and after 24 weeks of treatment were regarded as favorable responses to omalizumab therapy based on European Forum for Research and Education in Allergy and Airway Diseases (EUFOREA) expert board recommendations.^23^

### Sample collection

Blood and nasal secretion samples were obtained from patients at baseline and after 24 weeks of treatment. Nasal secretion samples were obtained by placing sinus sponge packs (Medtronic Xomed, Inc., Minneapolis, Minnesota, USA) in both nasal cavities for 5 min.^24^ NaCl solution (3 mL; 0.9%) was added to the nasal samples, then stored at 4 °C for 2 h to mobilize the nasal secretion. Furthermore, the sinus sponge was placed into the shaft of a syringe and centrifuged at 1500 g at 4 °C for 10 min. Blood eosinophil cells in 2 mL EDTA blood sample were automatically counted. Besides, serum samples were clotted at room temperature for 20 min, then centrifuged at 1500 g at 4 °C for 10 min. The samples were stored at −80 °C for further analysis.

### Cytokine, total IgE, and ECP levels in nasal secretion and serum samples

A total of 48 biomarkers were detected in nasal secretion and serum samples. The ImmunoCAP system (Phadia, Uppsala, Sweden) (total IgE and eosinophilic cationic protein [ECP] were tested), ELISA kits from R&D Systems (Minneapolis, USA) (periostin and myeloperoxidase, MPO), and Luminex 200 system (Luminex, Austin, Tex, USA). We detected T2 inflammatory biomarkers, including IL-3,^25^ IL-4, IL-5, IL-13, IL-25, IL-33, eotaxin, total IgE, periostin, ECP, granulocyte-macrophage colony-stimulating factor (GM-CSF) and CCL-19;^26^ T1 inflammatory biomarkers, including IL-2,^27^ IL-12p70, IL-15, IFN-γ, and IP-10 (CXCL-10);^28^ T3 inflammatory biomarkers, including IL-1 β, IL-8, IL-17, MPO, (CXCL)-1, CXCL-2, granulocyte colony-stimulating factor (G-CSF), and CCL-20; proinflammatory cytokines including IL-1α, IL-6, and tumor necrosis factor (TNF)-α, and anti-inflammatory cytokine, IL-10; We also detected chemokines, including chemokine (C–C motif) ligand (CCL)-2, CCL-3, CCL-4, CCL-5, remodeling factors, including epidermal growth factor (EGF), basic fibroblast growth factor (FGF-basic), platelet-derived growth factor (PDGF)-AA, PDGF-AB, transforming growth factor (TGF)-α and vascular endothelial growth factor (VEGF), cytotoxic mediators, granzyme B, and other biomarkers, including IL-1R α, IL-7, CD40L, FMS-like tyrosine kinase receptor 3 ligand (Flt-3 Ligand), interferon (IFN)-α, IFN-β, PD-L1, TNF-related apoptosis-inducing ligand (TRAIL). A BCA protein assay kit (Thermo Fisher Scientific, Rockford, Illinois USA) was used to normalize the concentration of biomarkers to the concentration of total protein.

### Statistical analysis

SPSS Statistics version 23.0 (SPSS, Inc, Chicago, USA) and GraphPad Prism 9 (GraphPad Software Inc., CA, USA) were used for all analyses. Values less than the limit of detection were deemed negative and assigned a value equal to half of the detection limit value for continuous analysis. The variables were treated as binomial variables since they had a high percentage (>33%) of negative values.^13^^,^^29^ The Kolmogorov‒Smirnov test was used to test the normality of the data distribution. The chi-square or Fisher's exact test was used for the dichotomous variable analysis. A Mann‒Whitney *U* 2-tailed test was used for between-group comparisons of continuous variables. The paired Wilcoxon signed rank test or McNemar test was used to compare biomarker concentrations at baseline and after 24 weeks of treatment. The correlation between biomarkers and the clinical manifestation improvement was assessed using Spearman's rank correlation analysis with the Benjamini‒Hochberg method for multiple testing. The ability of significantly different biomarkers to predict improvement in response to omalizumab treatment was determined using receiver operating characteristic (ROC) curves. The optimal cutoff value was determined using the Youden index. *P* < 0.05 was considered statistically significant.

## Results

### Study cohort

A total of 27 refractory CRSwNP patients with asthma were recruited in this study. However, 3 patients with incomplete examination data and 2 patients who did not meet the inclusion criteria were excluded from the study. Furthermore, 4 patients with incomplete nasal secretion samples and 6 patients with incomplete serum samples were excluded, and only 18 patients and 16 control subjects were ultimately included. The demographic and clinical characteristics of the enrolled patients are shown in Table 1. All refractory CRSwNP patients had a history of ESS and asthma comorbidity. Blood eosinophil count levels were higher than 150 cells/μL in all patients. Two patients reported headache on the injection day, but this symptom was relieved within 48 h.

**Table 1:** The baseline demographic and clinical characteristics of the patients. IQR, interquartile range

Characteristics	Patients (N = 18)	Control (N = 16)	*P*
Age (y), mean ± SD	46.44 (13.65)	40.61 (8.38)	0.168
Sex: Male, n (%)	11 (61.11)	10 (62.50)	>0.999
BMI (kg/m^2^), mean ± SD	24.62 (3.47)	24.84 (2.74)	>0.999
Patients with allergic rhinitis, n (%)	7 (38.89)	0 (0)	**0.008∗∗**
Serum total IgE (kU/L), median (IQR)	131.00 (77.65–259.75)	12.50 (9.01–43.42)	**<0.001∗∗∗**
Previous surgery/number, median (IQR)	1.0 (1.0–1.0)	/	/
Time from the last surgery to the treatment of omalizumab (month), median (IQR)	22.00 (15.50–57.25)	/	/
Dosages of omalizumab per month (mg), median (IQR)	300 (300–450)	/	/
Blood eosinophil count (cells/μL), median (IQR)	550.00 (225.00–1320.00)	/	/
Total endoscopic nasal polyp score, median (IQR), (range 0–8)	3.50 (2.00–5.00)	/	/
Total Lund-MacKay score, median (IQR), (range 0–24)	17.50 (14.00–19.25)	/	/
Total Lund-Kennedy score, median (IQR), (range 0–20)	10.00 (8.75–12.00)	/	/
SNOT-22 total score, median (IQR), (range 0–110)	47.00 (26.75–57.50)	/	/
VAS of nasal congestion score, median (IQR), (range 0–10)	7.10 (4.45–9.05)	/	/
VAS of runny nose score, median (IQR), (range 0–10)	6.95 (4.25–8.48)	/	/
VAS of sense of smell score, median (IQR), (range 0–10)	9.85 (6.93–10.00)	/	/
VAS of facial pressure score, median (IQR), (range 0–10)	1.70 (0.00–4.10)	/	/
Asthma control test score, median (IQR), (range 0–25)	21.00 (18.00–23.25)	/	/

The bold indicate the significant difference in two groups. ∗∗, *P* <0.01; ∗∗∗, *P* <0.001.

### Clinical assessment

**Fig. 1:**
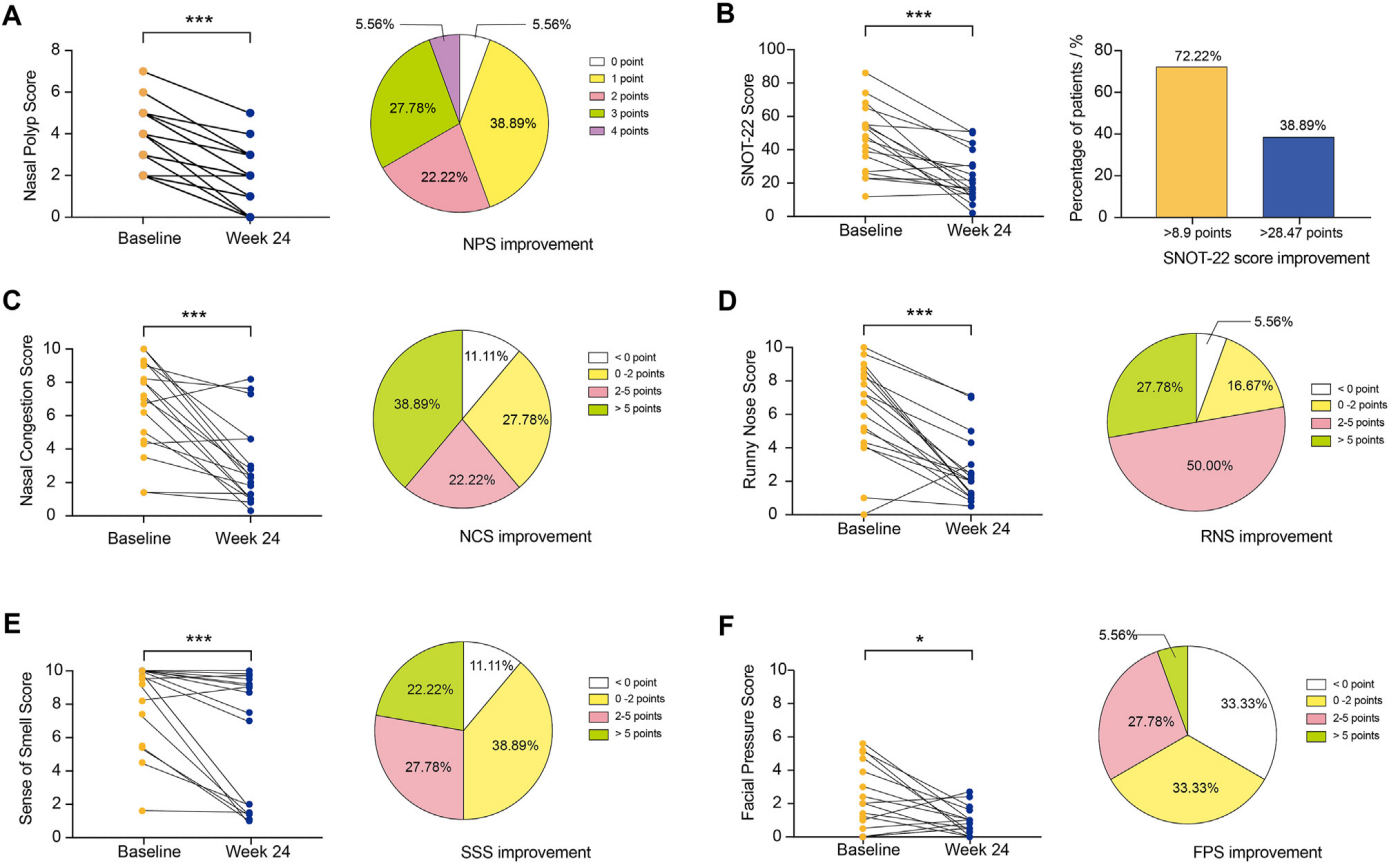
Clinical characteristics at baseline and at 24 weeks after omalizumab therapy. **(A)** Change in the nasal polyp score and distribution of improved points. **(B)** Change in the SNOT-22 score and distribution of satisfactory MCID and 3 MCIDs. **(C)** Change in the nasal congestion score and distribution of improved points. **(D)** Change in the runny nose score and distribution of improved points. **(E)** Change in the sense of smell score and distribution of improved points. **(F)** Change in the facial pain score and distribution of improved points. ∗, *P* < 0.05; ∗∗∗, *P* < 0.001.

Omalizumab significantly improved the endoscopic and radiographic results and patient-reported clinical symptoms and QOL, similar to a recently published study (Fig. 1).^17^ The NPS was significantly improved (median 2.0; IQR, 1.0–3.0, *P* < 0.001) by more than 2 points in approximately 55.56% of patients after the treatment of 24 weeks of omalizumab. However, 1 patient did not improve on this treatment, who probably present the mixed endotype inflammatory profile (Fig. 1A). A similar trend was observed in the LMS and LKS (all *P* < 0.001) after treatment. 55.56% of patients had an improvement of LMS higher than 6 points, while 4 patients showed LMS aggravation (median 7.5; IQR, −0.5-11.25). 61.12% of patients had an improvement of LKS lower than 5 points (median 4.0; IQR, 2.0–8.0).

The median (IQR) improvement in the SNOT-22 at week 24 was 21.00 points (4.75–36.75). Approximately 72.22% of patients exhibited an improvement of more than 8.9 points (>MCID) in the SNOT-22 score, while 38.89% of patients had an improvement in the SNOT-22 score of higher than 28.47 points (>3 MCIDs)^30^ after 24 weeks (Fig. 1B). The NCS, RNS, SSS, and FPS were significantly improved at week 24 (*P* < 0.001, *P* < 0.001, *P* < 0.001, and *P* = 0.021, respectively). The median change in the NCS for omalizumab from baseline to week 24 was 4.10 points (IQR, 0.60–6.40). Over 50% of patients exhibited an improvement in the NCS, RNS, and SSS, but not the FPS, of higher than 2 points (Fig. 1C–F). Omalizumab significantly improved the median of ACT scores from 21.00 points at baseline to 22.50 points at week 24. (*P* < 0.001).

### Biomarker analysis

A total of 47 biomarkers were detected in nasal secretion samples (except for IFN-β), while only 35 biomarkers were detected in serum samples (Table S1).

Firstly, we analyzed the concentration of biomarkers in serum in control subjects and patients with refractory CRSwNP. We found the T2 inflammatory cytokine IL-33, total IgE, and GM-CSF, chemokines such as CCL-3 and CCL-4 were significantly higher than in CRSwNP group (*P* < 0.001, <0.001, <0.001, <0.001, and 0.002, respectively). While the T3 inflammatory biomarker, G-CSF (*P* < 0.001), and remodeling factors, EGF and TGF-α were higher in control subjects than in refractory CRSwNP patients (*P* = 0.038 and < 0.001, respectively). At the same time, proinflammatory factor, IL-1α and TNF-α, and anti-inflammatory factor, IL-10, both higher in control subjects (*P* = 0.023, <0.001, <0.001, respectively). And other biomarkers like CD40L and PD-L1 were also significant higher in control subjects than that in refractory CRSwNP patients (all *P* were <0.001) (Table S1).

While many cytokines in nasal secretion showed higher expression in refractory CRSwNP, including T2 inflammatory cytokines, IL-3, IL-5, IL-25, IgE, and GM-CSF (*P* = 0.020, 0.009, 0.004, <0.001, and 0.002, respectively), and neutrophilic inflammatory cytokines, IL-8, MPO, and G-CSF (*P* = 0.031, 0.010, and 0.038, respectively) (also as T3 inflammatory biomarkers), IL-2, and IFN-γ (all *P* < 0.001*)* (also as T1 inflammatory biomarkers), than those in control subjects. There still are some proinflammatory cytokines, IL-6 (*P* = 0.026), and chemokine, CCL-2 (*P* = 0.004), remodeling factors, EGF and TGF-α (*P* < 0.001, 0.022, respectively), and other biomarkers including CD40L, IFN-α, PD-L1, and TRAIL were higher in refractory CRSwNP than in control subjects (*P* = 0.003, 0.002, 0.002, and 0.022, respectively). While the concentrations of IL-10 and PDGF-AB (*P* = 0.024 and 0.048) were higher in control subjects (Table S1).

**Fig. 2:**
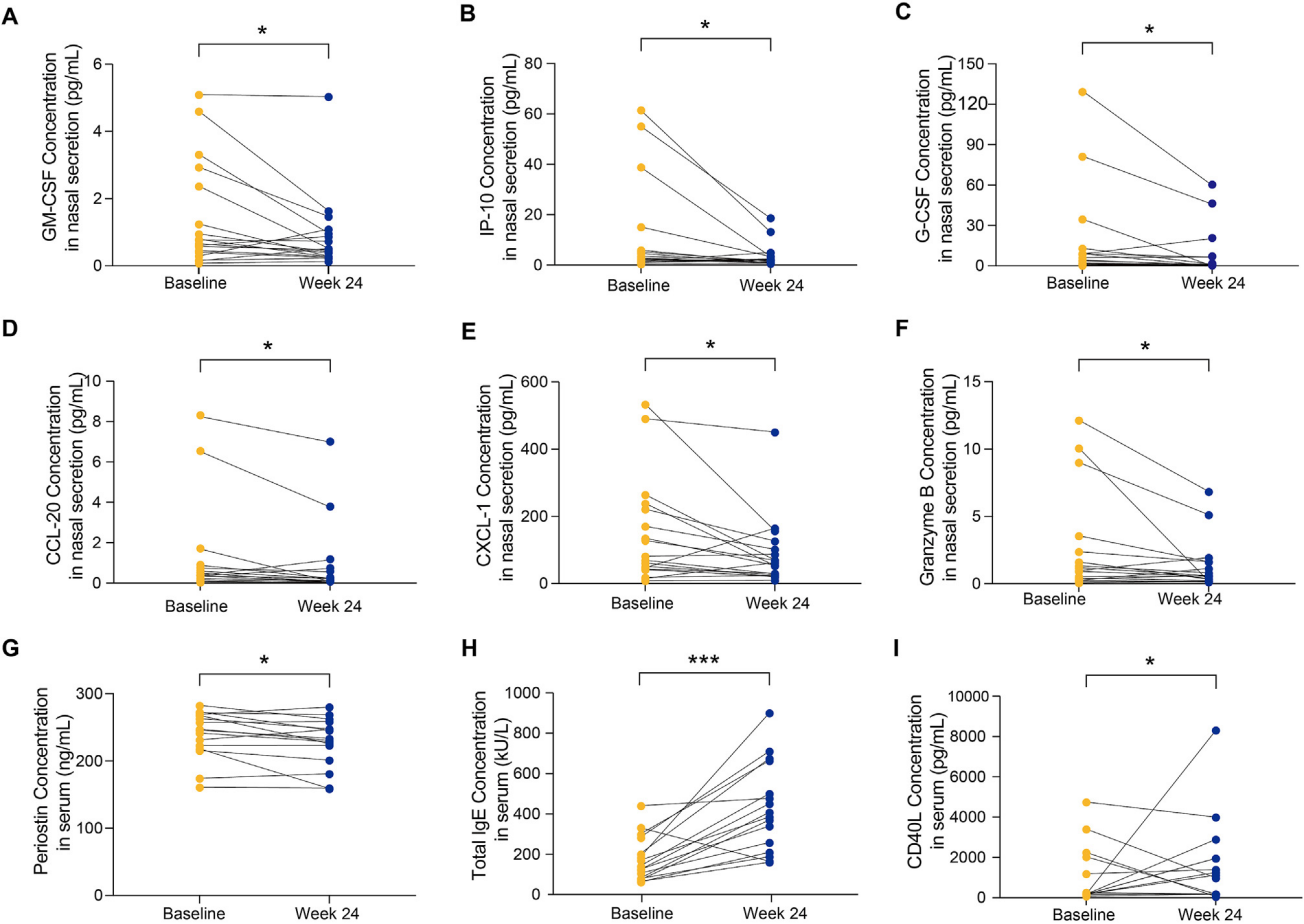
Levels of biomarkers at baseline and at 24 weeks after omalizumab treatment. The type 2 inflammatory biomarker, **(A)** GM-CSF, in nasal secretion. The type 1 inflammatory biomarker, **(B)** IP-10, in nasal secretion. The type 3 inflammatory biomarkers in nasal secretion, including **(C)** G-CSF, **(D)** CCL-20, and **(E)** CXCL-1. The cytotoxic mediator, **(F)** granzyme B in nasal secretion. The type 2 inflammatory biomarkers in serum, including **(G)** periostin and **(H)** total IgE. And other biomarker **(I)** CD40L level in serum. ∗, *P* < 0.05.

Secondly, we further explore the variations of biomarkers after the treatment of omalizumab. Typical T2 inflammatory biomarker, GM-CSF (*P* = 0.030) (Fig. 2A), T1 inflammatory biomarker, IP-10 (*P* = 0.016) (Fig. 2B), and T3 inflammatory biomarkers, G-CSF (*P* = 0.024) (Fig. 2C), CCL-20 (*P* = 0.023) (Fig. 2D), and CXCL-1 (*P* = 0.016) (Fig. 2E) in nasal secretions consistently decreased after omalizumab treatment. We also found cytotoxic mediators granzyme B decreased in nasal secretions after omalizumab treatment (*P* = 0.027) (Fig. 2F).

Furthermore, after the treatment of omalizumab treatment, we found the T2 inflammatory biomarker serum periostin (*P* = 0.025) (Fig. 2G) significantly decreased, and total IgE (*P* < 0.001) (Fig. 2H) increased. Besides, omalizumab therapy significantly increased serum CD40L levels (*P* = 0.039) (Fig. 2I). Omalizumab therapy did not affect other biomarkers in nasal secretion or serum samples.

### Correlation between biomarker levels and improvement in clinical manifestations

**Fig. 3:**
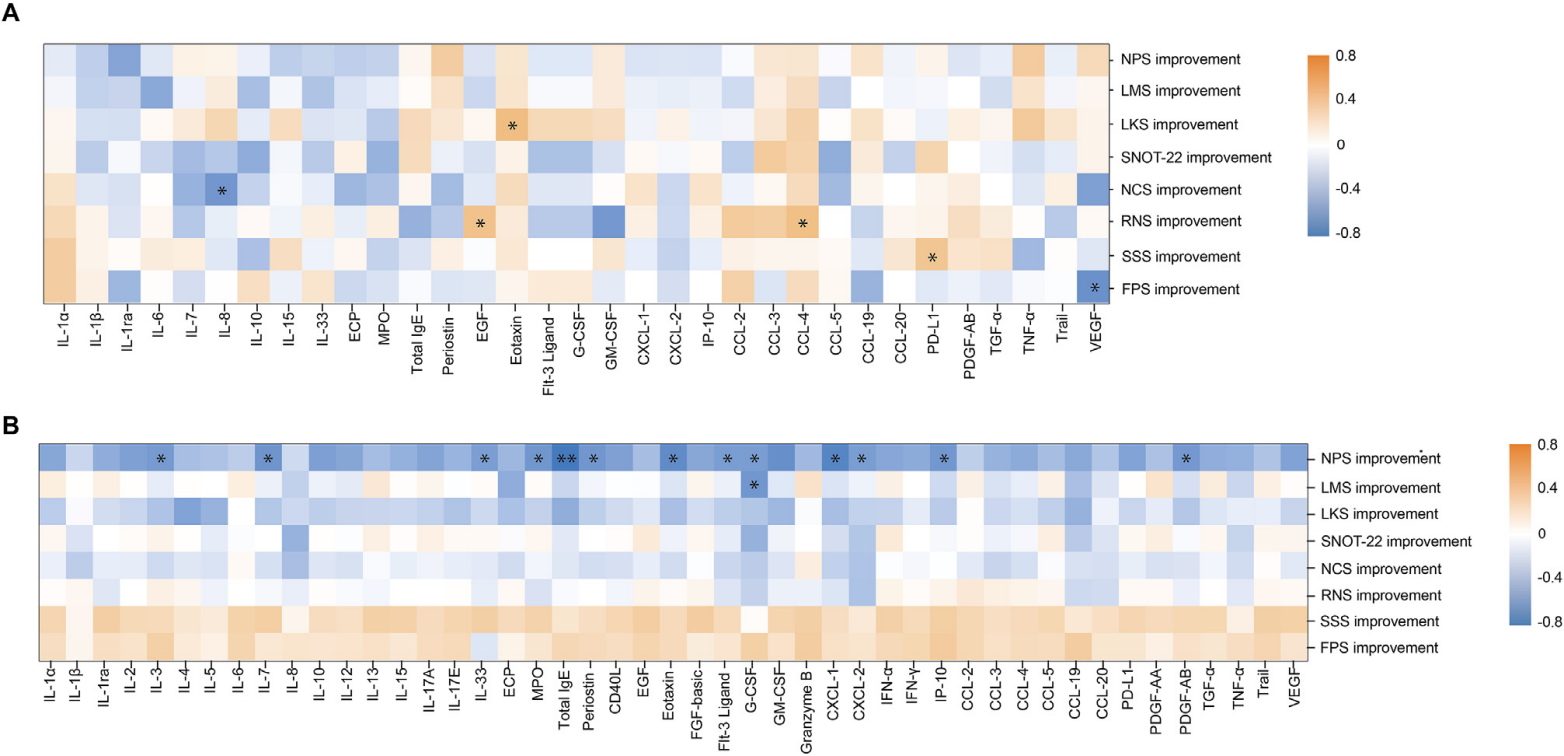
Spearman correlation analysis between clinical improvement and **(A)** levels of serum and **(B)** levels of nasal secretion biomarkers. The orange and blue areas indicate positive and negative correlations, respectively. The right side indicates the correlation coefficient. ∗, *P* < 0.05.

We further analyzed the correlation of the concentration of biomarkers at baseline and the improvement of clinical symtoms after the treatment of omalizumab. The concentration of serum eotaxin was positively correlated with improvement in the LKS (r = 0.5891, *P* = 0.016). The level of serum IL-8 was negatively correlated with improvement in the NCS (r = −0.5081, *P* = 0.044). The concentrations of serum EGF and CCL-4 were positively correlated with improvement in the RNS (r = 0.5479, *P* = 0.028 and r = 0.5752, *P* = 0.020, respectively). The concentration of GM-CSF in serum was negatively correlated with improvement in the RNS (r = −0.4985, *P* = 0.049). The serum PD-L1 and VEGF concentrations were positively and negatively correlated with improvement in the SSS and FPS, respectively (r = 0.5207, *P* = 0.039 and r = −0.5288, *P* = 0.035, respectively) (Fig. 3A).

The concentrations of many nasal secretion biomarkers, including IL-3, IL-7, IL-33, MPO, total IgE, periostin, eotaxin, Flt-3 ligand, G-CSF, GM-CSF, CXCL-1, CXCL-2, IP-10, and PDGF-AB, were significantly negatively correlated with improvement in the NPS. Additionally, the concentration of nasal secretion G-CSF was negatively correlated with improvement in the LMS (r = −0.5075, *P* = 0.032). The concentrations of IL-33, VEGF, TRAIL, and EGF were all positively correlated with improvement in the SSS (Fig. 3B).

### Prediction ability of biomarkers

An improvement higher than 8.9 points on the SNOT-22 or higher than 2 points on the VAS (NCS, RNS, SSS, and FPS) indicated favorable responses to type 2 biological therapy, according to EUFOREA expert recommendations ^(20)^. An NPS improvement higher than 2 points was considered a better response to omalizumab treatment. In this study, patients were divided into improved subgroup and none or less improved subgroup.

**Fig. 4:**
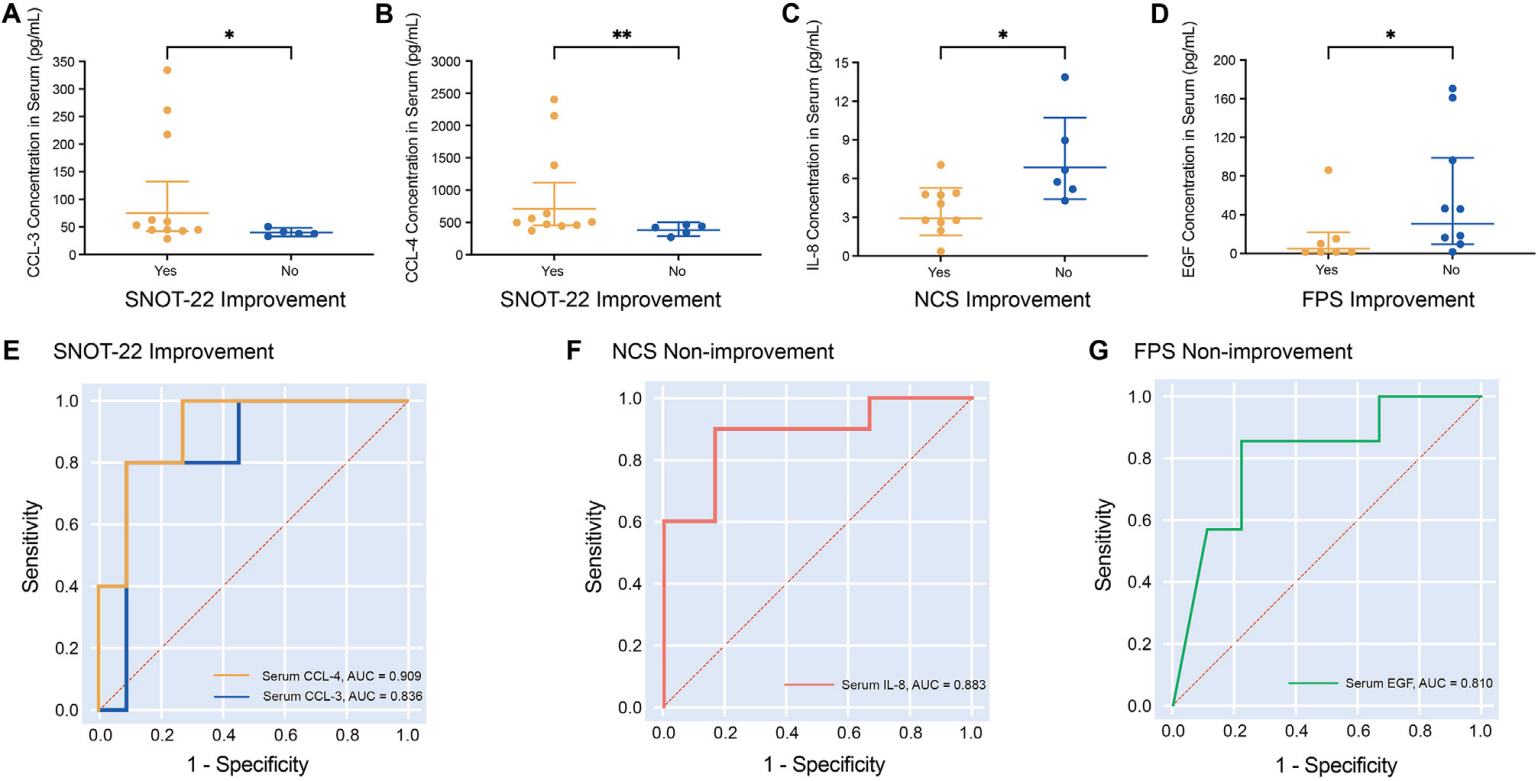
Differentially expressed biomarkers in serum in improved and non-improved subgroups based on favorable outcomes of different clinical symptoms. **(A**–**D)** Distinctly expressed biomarkers in serum. The error bars indicate the geometric mean with 95% CI. **(E)** ROC curve for the prediction of SNOT-22 score improvement based on serum CCL-3 and CCL-4 levels. **(F)** ROC curve for the prediction of NCS non-improvement based on serum IL-8 levels. **(G)** ROC curve for the prediction of FPS non-improvement based on serum EGF levels.

We analyzed the differences in the levels of serum biomarkers and nasal secretion biomarkers in the 2 subgroups, as shown in Tables S2-S7. The concentrations of serum CCL-3 and CCL-4 were significantly higher in the improved subgroup than in the non-improved subgroup based on improvement in the SNOT-22 score (*P* = 0.036 and *P* = 0.009, respectively) (Fig. 4A–B and Table S2). However, the concentrations of serum IL-8 and EGF were higher in the nonimproved subgroup than in the improved subgroup based on improvements in the NCS and FPS, respectively (*P* = 0.010 and *P* = 0.039, respectively) (Fig. 4C–D, Table S3, and Table S6).

Furthermore, ROC analysis was used to assess the ability of serum CCL-3 and serum CCL-4 to predict improvement in the SNOT-22 score. The AUCs of serum CCL-3 and CCL-4 for a favorable SNOT-22 outcome were excellent at 0.836 (95% CI, 0.621–1.000, *P* = 0.036) and 0.909 (95% CI, 0.762–1.000, *P* = 0.011), respectively. The sensitivity and specificity of serum CCL-3 and CCL-4 for predicting a favorable outcome according to the SNOT-22 score were 90.0% and 80.0%, respectively (optimal cutoffs: 84.350 pg/mL and 886.762 pg/mL, respectively) (Fig. 4E and Table S8). Moreover, a concentration of serum IL-8 higher than 5.020 ng/mL predicted NCS non-improvement, with 83.3% sensitivity and 90.0% specificity (AUC = 0.883, *P* = 0.013) (Fig. 4F and Table S8). Serum EGF higher than 15.750 pg/mL predicted FPS non-improvement with 77.8% sensitivity and 85.7% specificity (Fig. 4G and Table S8).

**Fig. 5:**
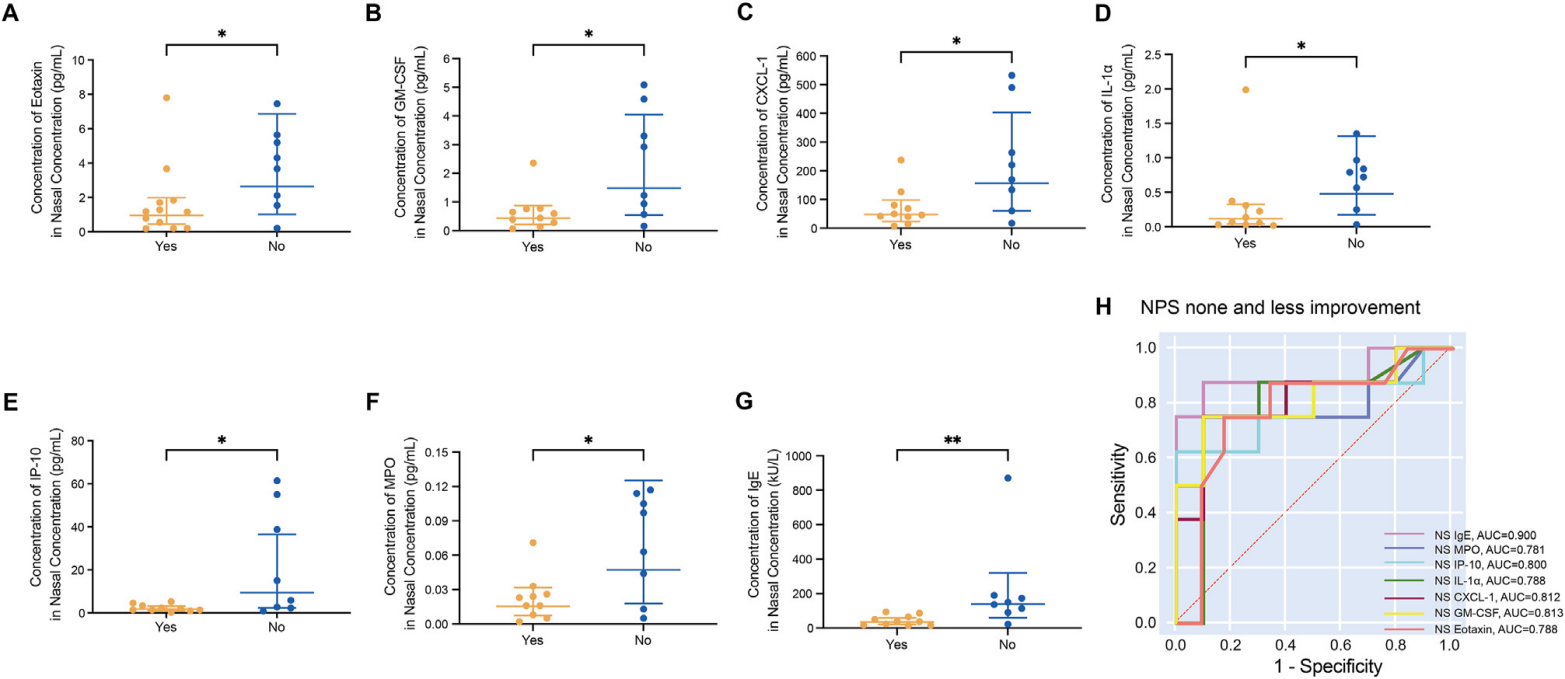
Differentially expressed biomarkers of nasal secretion in the none and less improved and better improved subgroups based on favorable outcomes of the NPS higher than 2 points. **(A**–**G)** Distinctly expressed biomarkers in nasal secretion. The error bars indicate the geometric mean with 95% CI. **(H)** ROC for the prediction of NPS none and less improved subgroups based on nasal secretion IgE, MPO, IP-10, IL-1a, CXCL-1, GM-CSF and eotaxin levels.

We also found that the nasal secretion concentrations of eotaxin, GM-CSF, CXCL-1, IP-10, IL-1α, MPO and IgE in the NPS nonimproved and less improved subgroups were significantly higher than those in the NPS better improved subgroup (*P* = 0.037, 0.027, 0.027, 0.034, 0.043, 0.046, and 0.003, respectively) (Fig. 5A–G and Table S7). According to the ROC analysis, the nasal concentrations of eotaxin, GM-CSF, CXCL-1, IP-10, IL-1α, MPO and IgE predicted the NPS none and less response with reliable sensitivity and specificity (Fig. 5H and Table S8). Among them, a nasal secretion IgE level higher than 88.555 kU/L predicted the NPS none and less improvement with 87.5% sensitivity and 90.0% specificity (AUC = 0.900, *P* = 0.004).

## Discussion

The current study demonstrated that omalizumab markedly ameliorated both subjective and objective evaluation endpoints in most Chinese patients with refractory CRSwNP with comorbid asthma, based on the evaluation of SNOT-22, VAS, NPS and LMS. Moreover, the significant change of levels of some inflammatory factors and chemokines in nasal secretion and serum were observed after omalizumab therapy. Most important of all, we found multiple chemokines as well as inflammatory, proinflammatory and growth factors in serum and nasal secretion could be used as suitable biomarkers to identify the favorable or poor response to omalizumab therapy, according to different clinical parameters.

In this study, the inclusion criteria for all CRSwNP patients followed the EUFOREA guidelines,^27^ and CRSwNP patients comorbid with asthma were selected to comply with the registered indication for omalizumab treatment for asthma based on the National Medical Products Administration in China. The monthly dosage of omalizumab costs approximately 500–2000 (CNY) after social insurance coverage in China, which is significantly less than the cost of dupilumab. Based on a signgle-center study in China, the median economic cost of omalizumab treatment for 24 weeks for patients with recurrent CRSwNP was 18,836 (CNY).^31^ Although regular biologics are promising treatments for uncontrolled CRSwNP or asthma, they are more expensive than conventional treatment. Therefore, the cost-effectiveness of multiple biologics should be compared to select the superior mAb in future studies.

Our real-world data in China further supported the impressive effectiveness and well tolerability of omalizumab at week 24 from phase 3 trials in Europe and the United States conducted by Gevaert, in which omalizumab also significantly improved endoscopic, clinical and patient-reported outcomes in severe CRSwNP patients.^16^ In contrast, a meta-analysis conducted by Oykhman^32^ showed that multiple biologics can significantly improve symptom-based outcomes, including the NCS, SNOT-22 score, and University of Pennsylvania Smell Identification Test (UPSIT) score, compared with the objective NPS. The difference could be due to different inclusion criteria. In addition, unlike this real-world study, the MCID of the SNOT-22 and VAS were used to assess favorable improvement, the subjective improvement in the mentioned meta-analysis was based on the change in the median difference.

Currently, the effect of biological therapy on inflammatory factors is unclear. Compared with a placebo, mepolizumab can increase serum levels of CCL-13, CCL-17, CCL-22, and eotaxin-1 in severe eosinophilic asthma patients, indicating the underlying compensatory mechanisms occurring in IL-5 blockade.^33^ A recent study indicated that serum inflammation-related proteins involved in chemotaxis, lymphocyte proliferation, and apoptosis are enriched in primary diffuse type 2 CRS after dupilumab treatment. Furthermore, no classical type 2 pathway cytokines were significantly activated in that study, but Th17, neutrophilic and regulator processes, including IL-10, were significantly activated.^34^ Unlike nasal mucosal biopsy, sample collection from blood and nasal secretion is less invasive, less costly, and easier to obtain and has higher patient compliance.^35^ In this study, changes in the inflammatory pathway were assessed by detecting markers in serum and nasal secretion samples. Omalizumab therapy decreased the concentrations of GM-CSF, G-CSF, granzyme B, CXCL-1, IP-10, and CCL-20 in nasal secretion and serum periostin samples. Although based on previous experience, G-CSF, CXCL-1, IP-10 and CCL-20 were typical type 1 or type 3 inflammatory biomarkers. In fact, mixed inflammatory patterns with the coexistence of eosinophil and neutrophil diversity were often present in CRSwNP tissues.^36^ Neutrophil infiltration was associated with eosinophil extracellular traps formation and charcot-leyden crystal, which were hallmarks in severe T2 CRSwNP.^37^^,^^38^ In addition, IL-8-mediated neutrophil recruitment was involved in the accumulation of eosinophils.^39^ Consequently, targeted biological therapy could potentially induce the change of biomarkers of different types of inflammation. We also noticed that omalizumab therapy increased serum CD40L and total IgE, indicating that blocking 1 inflammatory pathway activates another.^40^ In our study, we found serum CD40L was higher level in control subjects, and local CD40L was higher in refectory CRSwNP patients as previous study mentioned.^41^ Serum CD40L levels correlated with increased CD40L-positivs cell counts in the sinonasal mucosa, and it can impaired peripheral blood B cell function and enhanced the local inflammatory.^41^ After the treatment of anti-IgE, it may decrease the local inflammation and renovate the function of B cell, thus the serum CD40L was increased. Furthermore, it has been confirmed that upregulation of CD40L expression in mast cells could promote IgE synthesis.^42^ On the other side, Strohner suggested once omalizumab therapy is initiated, it was currently impossible to quantify free serum IgE as a means to confirm successful IgE elimination and guide future omalizumab dosing. Commercially available assays to quantify IgE recognize both free IgE and IgE molecules as part of omalizumab-IgE-complexes, leading to false IgE measurements.^43^

Suitable cost-effective biologics are needed to achieve optimal treatment outcomes. Several studies have explored potential biomarkers that can be used for specific targeted therapy. Serum osteoprotegerin (OPG) can predict a favorable outcome (AUC = 0.92) in response to dupilumab treatment specifically in patients with severe type 2 CRSwNP.^34^ In this study, serum eotaxin, EGF, CCL-4, and PD-L1 levels were positively correlated with improvements in the LKS, RNS, and SSS, respectively, at baseline. Furthermore, both serum IL-8 and VEGF levels were negatively correlated with improvements in the NCS and FPS. Interestingly, many nasal secretion biomarkers, including IL-3, IL-7, IL-33, MPO, total IgE, periostin, eotaxin, Flt-3 ligand, G-CSF, GM-CSF, CXCL-1, CXCL-2, IP-10, and PDGF-AB, were negatively correlated with improvement in the NPS. Normally, tissue eosinophil migration and accumulation are mostly influenced by chemokine network. It is also possible that the biological effect of some chemokines is by no means limited to the eosinophil migration and accumulation.^44^ We found serum CCL-3 (AUC = 0.836) and CCL-4 (AUC = 0.909) could effectively predict a favorable response to omalizumab based on the MCID of the SNOT-22 test. Similarly, Watanabe et al. showed that baseline serum CCL-3 and eotaxin levels can predict a good response to benralizumab treatment in patients with severe eosinophilic asthma.^45^ Recent research also revealed that CCL-4 was involved in eosinophil recruitment and that its expression was significantly upregulated in polyp tissue from eosinophilic CRSwNP patients.^46^ The latest study demonstrated higher tissue concentrations of CCL-3 and CCL-4 were observed in the endotype of high type 2 inflammation in CRS patients based on the inflammatory and remodeling factors, furthermore, the level of CCL-3 were positively correlated with the nasal polyp and CT score, respectively.^29^

The NPS and NCS are important aspects of the clinical assessment of the therapeutic efficacy of omalizumab. In this study, many biomarkers in nasal secretions (eotaxin, GM-CSF, CXCL-1, IP-10, IL-1α, MPO, IgE) or serum samples (IL-8) showed an association with poorer clinical outcomes. As is known that eotaxin, GM-CSF, and IgE are typical biomarkers for T2 CRSwNP and their concentrations are correlated with the degree of T2 inflammation.^29^ However, a real-world study demonstrated the level of IgE, IL-5 and ECP in the blood failed in serving as the predictive biomarkers for effectiveness of 3 kinds of biologics, including omalizumab. A recent study involving CD34 immunostaining probably illustrated the underlying mechanism, in which hypervascularity was present in the non-polyp mucosa and that hypovascularity was observed in T2 CRSwNP tissue.^47^ Therefore, it could be speculated that it might be difficult for systemic biologics to alleviate the effect of IgE and other type 2 inflammatory factors in local produced by eosinophils and Th2 cells due to the limitation of natural distribution of vascularity. Moreover, CXCL-1, IL-8 and MPO can recruit neutrophils in CRS and enhance neutrophilic inflammation.^48^ The abundances of IL-1α, IL-8 and CXCL-1 were also increased in non-eosinophilic CRSwNP tissues compared with eosinophilic tissues.^49^ The serum IL-8 level in asthma patients with poor corticosteroid responsiveness was significantly higher than that in healthy controls and the its degree of reduction also reflected the response to corticosteroids between uncontrolled and controlled asthma patients.^50^ Yoshikawa et al. demonstrated that IP-10 was overproduced in response to viral infection in patients with CRS comorbid with asthma, and it can also become intractable in treatment. All of the above findings imply that the variation in localized and systemic inflammation is related to the degree of alteration in the NPS or NCS.

Nevertheless, this study has some limitations. First, the relatively low number of included patients decreased the power of the statistical analysis. Second, all enrolled patients were from a single center. Therefore, a multicenter study with a larger sample size is needed to validate these findings. Third, patients with solo CRSwNP were not recruited due to the restriction of medical insurance policy in China, so comorbid asthma could be a confounding factor for evaluating the influence of omalizumab on patients.

## Conclusion

In summary, this study offered reliable evidence supporting the benefit of omalizumab in both subjective and objective outcomes in refractory CRSwNP patients with asthma. We not only observed the influence of anti-IgE on the variation in inflammatory mediators but also found a close association of endotype markers with the improvement in clinical parameters. More importantly, serum CCL-3 and CCL-4 were identified as ideal biomarkers for predicting a favorable SNOT-22 score in response to omalizumab in CRSwNP patients with asthma. Meanwhile, serum IL-8, as well as GM-CSF, CXCL-1, IP-10 and IgE in nasal secretion, could also predict the insufficient treatment response to omalizumab with good to excellent accuracy, based on the assessment of NCS and NPS. Larger-scale and multicenter studies will provide a comprehensive understanding of the nuance of CRSwNP endotypes, which will allow for more accurate selection of appropriate candidates to receive biologics.

## Abbreviations

ACT, asthma control test; AUC, area under the curve of the receiver operating characteristic; CRS, chronic rhinosinusitis; CRSsNP, chronic rhinosinusitis without nasal polyp; CRSwNP, chronic rhinosinusitis with nasal polyp; CCL, chemokine (C–C motif) ligand; CXCL, chemokine (C-X-C motif) ligand; ECP, eosinophilic cationic protein; EGF, epidermal growth factor; EMA, European Medicines Agency; ESS, endoscopic sinus surgery; EPOS, European Position Paper on Rhinosinusitis and Nasal Polyps; EUFOREA, European Forum for Research and Education in Allergy and Airway Diseases; FDA, U.S. Food and Drug Administration; FPS, facial pain score; G-CSF, granulocyte colony-stimulating factor (G-CSF); GINA, Global Initiative for Asthma guidelines; GM-CSF, granulocyte-macrophage colony-stimulating factor; IFN, interferon; LKS, Lund-Kennedy score; LMS, Lund-MacKay score; mAb, monoclonal antibody; MCID, minimal clinically important difference; MPO, myeloperoxidase; NPS, nasal polyp score; NCS, nasal congestion score; PD-L1, programmed cell death ligand 1; PDGF, platelet-derived growth factor; QALYs, quality-adjusted life years; QOL, quality of life; RESS, radical endoscopic sinus surgery; RNS, runny nose score; ROC, receiver operating characteristic; SNOT-22, 22-item sina-nasal outcome test; SSS, sense of smell score; TGF, transforming growth factor; Th2, T helper 2; TNF, tumor necrosis factor; TRAIT, TNF-related apoptosis-inducing ligand; UPSIT, University of Pennsylvania Smell Identification Test; VAS, visual analogue scale; VEGF, vascular endothelial growth factor.

## Author contribution

YS, MZ, CL, XW and LZ conceived, collected the clinical data, and drafted the manuscript. LZ and XW supervised and conceptualized the study and were involved in the important revision of the manuscript. YS, MZ, and SG performed the data analysis. YS, MZ and PW analyzed the endotype data under the guidance of YZ. All the authors have read and approved the final version of the manuscript.

## Consent for publication

All authors have read the final manuscript and agreed to publication of the work.

## Ethics approval

This study was approved by the Ethics Committee of Beijing Tongren Hospital and Chinese Clinical Trail Registry. And written informed consent was obtained from all participants.

## Funding

This project was supported by the National Key R&D Program of China (2022YFC2504100), the program for the Changjiang Scholars and Innovative Research Team (IRT13082), the 10.13039/501100001809National Natural Science Foundation of China (82171110, 82000962, and 81970852), the CAMS Innovation Fund for Medical Sciences (2019-I2M-5–022), the Capital's funds for health improvement and research (2022-1-1091), the Beijing Natural Science Foundation (7222024), the Beijing New-star Plan of Science and Technology (20220484226), the Beijing Hospitals Authority Youth Programme (QML20230201), the Public Welfare Development and Reform Pilot Project (2019–10), the 10.13039/501100009592Beijing Municipal Science & Technology Commission (Z211100002921057) and the Special Funds for the Construction of High-level Public 10.13039/100018696Health Technical Talents (Lingjunrencai-01-08 and Lingjunrencai-02-09).

## Declaration of competing interest

All the authors declare that they have no conflicts of interest related to the contents of this work.

## Availability of data and material

The corresponding author will provide the datasets that support the study's findings on reasonable request.
