# Supplementary material for: Predicting the effectiveness of omalizumab in patients with refractory chronic rhinosinusitis with nasal polyps comorbid with asthma based on inflammatory biomarkers
Source: World Allergy Organ J. 2024 Dec 12;18(1):101009. doi: 10.1016/j.waojou.2024.101009 (PMC11700293; doi:10.1016/j.waojou.2024.101009)
Supplement: Multimedia component 1 [file mmc1.docx]

**Supplementary Figure**

**Figure S1.** Patient and sample disposition.


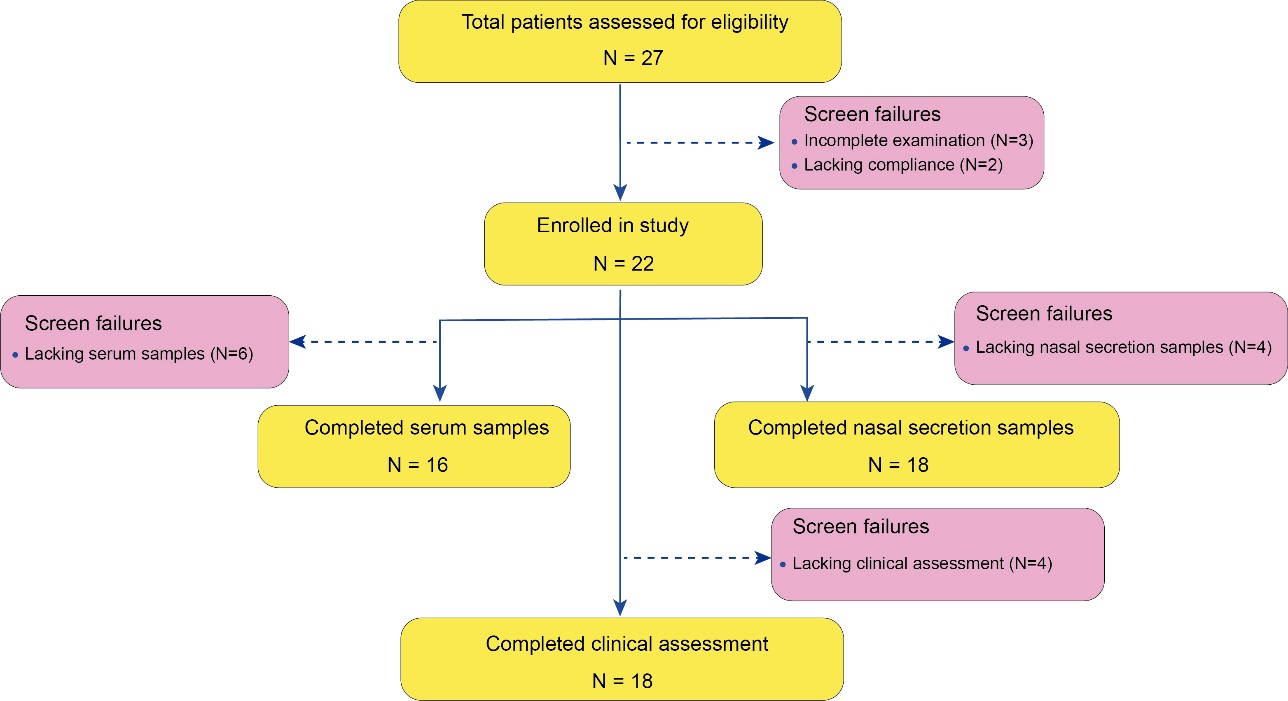


**Supplementary Tables**

**Table S1.** The concentration of biomarkers in control subjects and patients with refractory CRSwNP treated with omalizumab at baseline and week 24.

| Characteristics |  | Serum | | | | |  | Characteristics |  | Nasal secretion | | | | |
| --- | --- | --- | --- | --- | --- | --- | --- | --- | --- | --- | --- | --- | --- | --- |
|  |  | Control subjects | CRSwNP | | *P*1 | *P*2 |  |  |  | Control subjects | CRSwNP | | *P*1 | *P*2 |
|  |  |  | Baseline | Week 24 |  |  |  |  |  |  | Baseline | Week 24 |  |  |
| **T2 inflammatory biomarkers** | | | | | | | | | | | | | | |
| IL-3, pg/mL  median (IQR) |  | - | - | - | - | - |  | IL-3, $\mu$g/mL  median (IQR) |  | 0.16  (0.03 – 0.24) | 0.32  (0.08 – 1.15) | 0.25  (0.10 – 0.53) | **0.020*** | 0.610 |
| IL-4, pg/mL  median (IQR) |  | - | - | - | - | - |  | IL-4, $\mu$g/mL  median (IQR) |  | 0.02  (0.01 – 0.03) | 0.02  (0.01 – 0.07) | 0.01  (0.03 – 0.07) | 0.264 | 0.975 |
| IL-5, pg/mL  median (IQR) |  | - | - | - | - | - |  | IL-5, $\mu$g/mL  median (IQR) |  | 0.03  (0.01 – 0.05) | 0.08  (0.03 – 0.12) | 0.05  (0.02 – 0.09) | **0.009*** | 0.325 |
| IL-13, pg/mL  median (IQR) |  | - | - | - | - | - |  | IL-13, $\mu$g/mL  median (IQR) |  | 0.68  (0.13 – 0.99) | 0.44  (0.07 – 1.36) | 0.37  (0.14 – 0.57) | 0.942 | 0.442 |
| IL-25, pg/mL  median (IQR) |  | - | - | - | - | - |  | IL-25, $\mu$g/mL  median (IQR) |  | 0.05  (0.01 – 0.11) | 0.16  (0.06 – 0.75) | 0.15  (0.04 – 0.82) | **0.004**** | 0.742 |
| IL-33, pg/mL  median (IQR) |  | 3.28  (2.96 – 3.75) | 6.12  (4.24 – 7.78) | 6.96  (4.30 – 10.64) | **<0.001**  ******* | 0.659 |  | IL-33, $\mu$g/mL  median (IQR) |  | 0.53  (0.14 – 1.27) | 0.82  (0.41 – 1.69) | 0.46  (0.33 – 1.22) | 0.515 | 0.369 |
| Eotaxin, pg/mL  median (IQR) |  | 164.90  (148.96 – 181.28) | 128.16  (96.22 – 175.42) | 145.76  (125.48 – 202.50) | 0.065 | 0.348 |  | Eotaxin, $\mu$g/mL  median (IQR) |  | 1.21  (0.48 – 2.32) | 1.62  (0.73 – 4.53) | 1.28  (0.84 – 2.80) | 0.176 | 0.640 |
| Total IgE, kU/L  median (IQR) |  | 12.50  (9.01 – 43.42) | 131.00  (77.65 – 259.75) | 394.50  (220.00 – 621.25) | **<0.001**  ******* | **<0.001**  ******* |  | Total IgE, kU/L  median (IQR) |  | 2.85  (2.53 – 5.36) | 75.57  (23.15 – 129.63) | 77.42  (46.69 – 177.75) | **<0.001***** | 0.369 |
| ECP, ug/L  median (IQR) |  | 4.69  (2.79 – 14.08) | 6.23  (3.44 – 11.63) | 10.23  (6.45 – 14.00) | 0.224 | 0.252 |  | ECP, g/L  median (IQR) |  | 0.16  (0.01 – 0.26) | 0.12  (0.02 – 0.35) | 0.09  (0.02 – 0.19) | 0.734 | 0.966 |
| Periostin, ng/mL  median (IQR) |  | / | 245.94  (218.49 – 268.10) | 231.32  (206.04 – 255.19) | / | **0.025*** |  | Periostin, mg/mL  median (IQR) |  | / | 2.48  (1.07 – 8.68) | 2.12  (1.28 – 5.3) | / | 0.832 |
| GM-CSF, pg/mL  median (IQR) |  | 7.96  (5.41 – 8.82) | 21.64  (16.12 – 25.84) | 18.26  (12.26 –25.82) | **<0.001**  ******* | 0.316 |  | GM-CSF, $\mu$g/mL  median (IQR) |  | 0.23  (0.10 – 0.41) | 0.70  (0.36 – 2.50) | 0.48  (0.26 – 0.98) | **0.002**** | **0.030*** |
| CCL-19, pg/mL  median (IQR) |  | 102.32  (87.88 – 138.38) | 89.98  (62.24 – 118.18) | 76.28  (68.32 – 83.30) | 0.092 | 0.144 |  | CCL-19, $\mu$g/mL  median (IQR) |  | 1.07  (0.39 – 2.17) | 2.00  (1.01 – 6.27) | 1.08  (0.68 – 3.34) | 0.067 | 0.246 |
| **T1 inflammatory biomarkers** | | | | | | | | | | | | | | |
| IL-2, pg/mL  median (IQR) |  | - | - | - | - | - |  | IL-2, $\mu$g/mL  median (IQR) |  | 0.03  (0.01 – 0.06) | 0.13  (0.04 – 0.40) | 0.09  (0.04 – 0.18) | **<0.001***** | 0.640 |
| IL-12p70, pg/mL  median (IQR) |  |  | - | - | - | - |  | IL-12p70, $\mu$g/mL  median (IQR) |  | 0.19  (0.06 – 0.27) | 0.08  (0.03 – 0.26) | 0.04  (0.02 – 0.19) | 0.317 | 0.130 |
| IL-15, pg/mL  median (IQR) |  | 2.46  (1.72 – 2.97) | 1.78  (1.56 – 2.64) | 1.94  (1.34 – 2.94) | 0.135 | 0.843 |  | IL-15, $\mu$g/mL  median (IQR) |  | 0.03  (0.01 – 0.04) | 0.05  (0.02 – 0.15) | 0.05  (0.01 – 0.06) | 0.058 | 0.773 |
| IFN-γ, pg/mL  median (IQR) |  | - | - | - |  | - |  | IFN-γ, $\mu$g/mL  median (IQR) |  | 0.01  (0.01 – 0.01) | 0.26  (0.11- 0.69) | 0.19  (0.07 – 0.39) | **<0.001***** | 0.442 |
| IP-10, pg/mL  median (IQR) |  | 112.31  (91.62 – 133.72) | 100.74  (33.39 – 73.93) | 108.84  (24.04 – 69.62) | 0.689 | 0.934 |  | IP-10, $\mu$g/mL  median (IQR) |  | 2.59  (1.21 – 4.00) | 2.53  (1.37 – 8.12) | 1.83  (0.81 – 3.33) | 0.573 | **0.016*** |
| **T3 inflammatory biomarkers** | | | | | | | | | | | | | | |
| IL-1β, pg/mL  median (IQR) |  | 6.61  (2.84 – 6.74) | 5.62  (2.84 – 6.76) | 5.92  (3.38 – 6.96) | 0.635 | 0.531 |  | IL-1β, $\mu$g/mL  median (IQR) |  | 0.13  (0.05 – 0.24) | 0.20  (0.02 – 0.62) | 0.08  (0.03 – 0.46) | 0.165 | 0.387 |
| IL-8, pg/mL  median (IQR) |  | 8.15  (6.37 – 11.76) | 9.46  (5.54 – 12.88) | 8.68  (5.84 – 10.86) | 0.948 | 0.706 |  | IL-8, $\mu$g/mL  median (IQR) |  | 11.12  (1.93 – 20.45) | 21.11  (7.08 – 65.05) | 9.69  (2.77 – 26.93) | **0.031*** | 0.265 |
| IL-17, pg/mL  median (IQR) |  | - | - | - |  | - |  | IL-17, $\mu$g/mL  median (IQR) |  | 0.04  (0.01 – 0.09) | 0.04  (0.02 – 0.11) | 0.03  (0.01 – 0.10) | 0.568 | 0.678 |
| MPO, ng/mL  median (IQR) |  | 288.35  (224.10 – 701.25) | 192.41  (134.35 – 360.04) | 413.53  (133.57 – 500.47) | 0.066 | 0.376 |  | MPO, mg/mL  median (IQR) |  | 0.01  (0.01 – 0.01) | 0.03  (0.01 – 0.08) | 0.02  (0.01 – 0.07) | **0.010*** | 0.890 |
| CXCL-1, pg/mL  median (IQR) |  | 103.84  (73.80 – 159.20) | 100.74  (66.78 – 145.86) | 108.84  (48.08 – 139.24) | 0.583 | 0.934 |  | CXCL-1, $\mu$g/mL  median (IQR) |  | 83.77  (8.93 – 251.82) | 74.22  (41.52 – 224.36) | 57.24  (24.43 – 106.72) | 0.696 | **0.016*** |
| CXCL-2, pg/mL  median (IQR) |  | 471.28  (305.62 – 770.92) | 520.58  (409.42 – 956.52) | 559.40  (435.56 – 734.80) | 0.465 | 1.000 |  | CXCL-2, $\mu$g/mL  median (IQR) |  | 1.96  (0.55 – 3.81) | 3.39  (1.68 – 7.05) | 1.64  (0.79 – 4.29) | 0.126 | 0.284 |
| G-CSF, pg/mL  median (IQR) |  | 323.60  (29.20 – 41.02) | 67.38  (43.04 – 92.68) | 75.90  (55.52 – 87.18) | **<0.001**  ******* | 0.562 |  | G-CSF, $\mu$g/mL  median (IQR) |  | 1.26  (0.20 – 3.93) | 3.69  (1.02 – 9.80) | 1.02  (0.50 – 7.01) | **0.038*** | **0.024*** |
| CCL-20, pg/mL  median (IQR) |  | 14.82  (13.34 – 16.08) | 11.30  (4.58 – 17.28) | 9.00  (7.18 – 12.04) | 0.097 | 0.421 |  | CCL-20, $\mu$g/mL  median (IQR) |  | 0.17  (0.06 – 0.44) | 0.39  (0.17 – 0.80) | 0.16  (0.08 – 0.63) | 0.0538 | **0.023*** |
| **Proinflammatory cytokines** | | | | | | | | | | | | | | |
| IL-1α, pg/mL  median (IQR) |  | 17.32  (15.48 – 20.86) | 15.52  (11.06 – 17.14) | 15.04  (9.74 – 17.36) | **0.023*** | 0.893 |  | IL-1α, $\mu$g/mL  median (IQR) |  | 0.54  (0.18 – 0.90) | 0.28  (0.06 – 0.80) | 0.26  (0.14 – 0.44) | 0.286 | 0.932 |
| IL-6, pg/mL  median (IQR) |  | 7.12  (5.94 - 9.08) | 13.62  (7.22 – 15.16) | 9.40  (7.22 – 15.16) | 0.123 | 0.749 |  | IL-6, $\mu$g/mL  median (IQR) |  | 0.23  (0.06 – 0.49) | 0.57  (0.24 – 1.60) | 0.27  (0.14 – 0.86) | **0.026*** | 0.284 |
| TNF-α, pg/mL  median (IQR) |  | 7.66  (5.96 – 9.88) | 4.04  (1.12 – 4.80) | 4.80  (2.96 – 8.08) | **<0.001**  ******* | 0.382 |  | TNF-α, $\mu$g/mL  median (IQR) |  | 0.05  (0.01 – 0.10) | 0.20  (0.09 – 0.54) | 0.12  (0.03 – 0.32) | **<0.001***** | 0.074 |
| **Anti-inflammatory cytokines** | | | | | | | | | | | | | | |
| IL-10, pg/mL  median (IQR) |  | 142.40  (104.78 – 1645.64) | 31.14  (28.52 – 39.02) | 27.18  (17.86 – 42.92) | **<0.001**  ******* | 0.397 |  | IL-10, $\mu$g/mL  median (IQR) |  | 5.43  (1.65 – 8.83) | 1.24  (0.56 – 5.49) | 1.24  (0.52 – 2.39) | **0.024*** | 0.375 |
| **Chemokines** | | | | | | | | | | | | | | |
| CCL-2, pg/mL  median (IQR) |  | 256.84  (219.84 – 332.80) | 250.00  (133.56 – 333.66) | 266.42  (235.74 – 317.40) | 0.741 | 0.597 |  | CCL-2, $\mu$g/mL  median (IQR) |  | 0.65  (0.24 – 1.25) | 2.20  (0.94 – 2.54) | 0.87  (0.56 – 1.99) | **0.004**** | 0.330 |
| CCL-3, pg/mL  median (IQR) |  | 15.20  (15.20 – 15.20) | 90.02  (77.30 – 124.08) | 93.88  (70.86 – 131.96) | **<0.001**  ******* | 0.495 |  | CCL-3, $\mu$g/mL  median (IQR) |  | 0.40  (0.02 – 0.56) | 0.45  (0.16 – 1.50) | 0.28  (0.15 – 0.93) | 0.077 | 0.958 |
| CCL-4, pg/mL  median (IQR) |  | 592.70  (476.14 – 935.32) | 941.36  (851.02 – 1238.34) | 976.60  (576.04 –1405.06) | **0.002**** | 0.762 |  | CCL-4, $\mu$g/mL  median (IQR) |  | 3.54  (1.04 – 5.80) | 4.87  (2.48 – 13.70) | 2.92  (1.77 – 11.81) | 0.874 | 0.602 |
| CCL-5, ng/mL  median (IQR) |  | 35.42  (25.40 – 50.00) | 43.02  (28.04 – 56.68) | 42.04  (28.90 – 53.70) | 0.369 | 0.348 |  | CCL-5, $m$g/mL  median (IQR) |  | 0.80  (0.33 – 2.77) | 1.88  (0.48 – 6.32) | 1.48  (0.63 – 6.42) | 0.331 | 0.468 |
| **Remodeling factors** | | | | | | | | | | | | | | |
| EGF, pg/mL  median (IQR) |  | 94.22  (64.64 – 125.74) | 32.88  (3.04– 182.46) | 58.74  (27.98 – 207.26) | **0.038*** | 0.600 |  | EGF, $\mu$g/mL  median (IQR) |  | 0.02  (0.01 – 0.02) | 2.82  (1.69 – 9.27) | 2.30  (1.29 – 6.27) | **<0.001***** | 0.393 |
| FGF-basic, pg/mL  median (IQR) |  | 3.94  (3.94 – 4.68) | 3.42  (0.64 – 4.76) | 3.42  (3.22 – 6.62) | 0.933 | 0.625 |  | FGF-basic, $\mu$g/mL  median (IQR) |  | 0.33  (0.08 – 1.39) | 0.33  (0.10 – 0.95) | 0.33  (0.12 – 0.44) | 0.965 | 0.710 |
| PDGF-AA, pg/mL  median (IQR) |  | - | - | - | - | - |  | PDGF-AA, $\mu$g/mL  median (IQR) |  | 1.86  (0.63 – 3.48) | 2.75  (1.08 – 4.77) | 1.60  (1.02 – 2.79) | 0.443 | 0.417 |
| PDGF-AB, pg/mL  median (IQR) |  | 4274.80  (3637.68 – 55444.90) | 2960.70  (1745.24 – 4031.50) | 1830.484  (2280.40 – 4551.72) | 0.024 | 0.597 |  | PDGF-AB, $\mu$g/mL  median (IQR) |  | 0.18  (0.05 – 0.29) | 0.06  (0.02 – 0.12) | 0.03  (0.02 – 0.13) | **0.048*** | 0.495 |
| TGF-α, pg/mL  median (IQR) |  | 14.48  (13.64 – 18.12) | 8.50  (3.90 – 12.70) | 10.78  (4.28 – 13.08) | **<0.001**  ******* | 0.659 |  | TGF-α, $\mu$g/mL  median (IQR) |  | 0.17  (0.09 – 0.30) | 0.53  (0.13 – 1.37) | 0.43  (0.21 – 0.70) | **0.022*** | 0.890 |
| VEGF, pg/mL  median (IQR) |  | 164.42  (108.68 – 194.62) | 116.16  (98.12–156.32) | 133.86  (96.06 – 201.70) | 0.070 | 0.348 |  | VEGF, $\mu$g/mL  median (IQR) |  | 3.64  (0.99 – 5.03) | 2.66  (1.78 – 6.19) | 2.25  (1.26 0 4.47) | 0.806 | 0.468 |
| **Cytotoxic mediators** | | | | | | | | | | | | | | |
| Granzyme B, pg/mL  median (IQR) |  | - | - | - | - | - |  | Granzyme B, $\mu$g/mL  median (IQR) |  | 0.55  (0.16 – 1.10) | 1.05  (0.30 – 2.65) | 0.56  (0.19 – 1.60) | 0.099 | **0.027*** |
| **Other biomarkers** | | | | | | | | | | | | | | |
| IL-1 ra, pg/mL  median (IQR) |  | 483.92  (424.43 - 573.22) | 395.78  (324.70 – 468.28) | 436.02  (345.22 – 548.38) | 0.073 | 0.562 |  | IL-1 ra, $\mu$g/mL  median (IQR) |  | 93.42  (17.05 – 255.29) | 69.78  (24.36 – 277.52) | 58.71  (26.92 – 175.93) | 0.937 | 0.602 |
| IL-7, pg/mL  median (IQR) |  | 12.48  (9.58 – 17.44) | 12.48  (9.60 – 17.44) | 12.48  (9.52 – 16.86) | 0.318 | 0.487 |  | IL-7, $\mu$g/mL  median (IQR) |  | 0.12  (0.05 – 0.27) | 0.12  (0.05 – 0.28) | 0.07  (0.04 – 0.13) | 0.370 | 0.193 |
| CD40L, pg/mL  median (IQR) |  | 1707.58  (1080.05 – 2669.50) | 164.96  (164.96 –1801.46) | 1034.26  (164.96 – 1940.52) | **<0.001**  ******* | **0.039*** |  | CD40L, $\mu$g/mL  median (IQR) |  | 12.48  (0.55 – 16.12) | 24.02  (7.09 – 82.63) | 18.02  (7.49 – 33.63) | **0.003**** | 0.304 |
| Flt-3 Ligand, pg/mL  median (IQR) |  | 99.96  (93.56 – 106.32) | 87.50  (74.56 – 103.32) | 88.04  (68.54 – 107.48) | 0.144 | 0.860 |  | Flt-3 Ligand, $\mu$g/mL  median (IQR) |  | 1.08  (0.19 – 1.68) | 1.00  (0.37 – 2.27) | 0.62  (0.30 – 1.43) | 0.517 | 0.229 |
| IFN-α, pg/mL  median (IQR) |  | - | - | - | - | - |  | IFN-α, $\mu$g/mL  median (IQR) |  | 0.05  (0.01 – 0.09) | 0.13  (0.04 – 0.53) | 0.14  (0.05 – 0.27) | **0.002**** | 0.758 |
| IFN-$\beta$, pg/mL  median (IQR) |  | - | - | - | - | - |  | IFN-$\beta$, $\mu$g/mL  median (IQR) |  | - | - | - | - | - |
| PD-L1, pg/mL  median (IQR) |  | 102.14  (84.74 – 121.98) | 27.42  (24.30 – 56.12) | 41.44  (24.30 – 76.04) | **<0.001**  ******* | 0.156 |  | PD-L1, $\mu$g/mL  median (IQR) |  | 0.48  (0.14 – 0.76) | 1.37  (0.67 – 4.97) | 1.30  (0.47 – 2.72) | **0.002**** | 1.000 |
| TRAIL, pg/mL  median (IQR) |  | 38.02  (24.69 – 48.60) | 34.94  (20.24 – 49.04) | 39.28  (29.50 – 43.90) | 0.759 | 0.495 |  | TRAIL, $\mu$g/mL  median (IQR) |  | 2.32  (0.99 – 7.41) | 7.28  (4.19 – 31.36) | 5.26  (3.07 – 14.74) | **0.022*** | 0.081 |

*P*_1_, the difference between control subjects and patients with refractory CRSwNP at baseline. *P*_2_, the difference between patients with refractory CRSwNP at baseline and week 24. *, *P* < 0.05; **, *P* < 0.01; ***, *P* < 0.001.

.

**Table S2.** The difference of biomarkers based on the SNOT-22 improvement more than 8.9 points.

| Characteristics |  | Serum (N = 16) | | |  | Characteristics | Nasal secretion (N = 18) | | |
| --- | --- | --- | --- | --- | --- | --- | --- | --- | --- |
|  |  | Yes | No | *P* value |  |  | Yes | No | *P* value |
| IL-1α, pg/mL  median (IQR) |  | 15.52  (12.62 – 17.36) | 15.52  (5.12 – 18.18) | 0.492 |  | IL-1α, $\mu$g/mL  median (IQR) | 0.31  (0.04 – 0.78) | 0.25  (0.18 – 0.88) | 0.687 |
| IL-1β, pg/mL  median (IQR) |  | 5.52  (3.18 – 6.14) | 6.14  (2.06 – 7.58) | 0.764 |  | IL-1β, $\mu$g/mL  median (IQR) | 0.16  (0.02 – 0.57) | 0.42  (0.07 – 1.79) | 0.516 |
| IL-1 ra, pg/mL  median (IQR) |  | 377.96  (313.82 – 469.68) | 424.84  (347.78 – 499.02) | 0.661 |  | IL-1 ra, $\mu$g/mL  median (IQR) | 41.53  (16.71 – 298.54) | 79.17  (47.85 – 293.32) | 0.399 |
| IL-2, pg/mL  median (IQR) |  | - | - | - |  | IL-2, $\mu$g/mL  median (IQR) | 0.13  (0.02 – 0.33) | 0.14  (0.11 – 0.43) | 0.503 |
| IL-3, pg/mL  median (IQR) |  | - | - | - |  | IL-3, $\mu$g/mL  median (IQR) | 0.32  (0.04 – 1.28) | 0.33  (0.22 – 0.89) | 0.924 |
| IL-4, pg/mL  median (IQR) |  | - | - | - |  | IL-4, $\mu$g/mL  median (IQR) | 0.02  (0.00 – 0.07) | 0.03  (0.01 – 0.11) | 0.503 |
| IL-5, pg/mL  median (IQR) |  | - | - | - |  | IL-5, $\mu$g/mL  median (IQR) | 0.07  (0.02 – 0.10) | 0.12  (0.06 – 0.15) | 0.200 |
| IL-6, pg/mL  median (IQR) |  | 13.62  (7.22 – 13.62) | 13.62  (2.64 – 25.18) | 0.800 |  | IL-6, $\mu$g/mL  median (IQR) | 0.61  (0.09 – 1.44) | 0.53  (0.44 – 2.80) | 0.503 |
| IL-7, pg/mL  median (IQR) |  | 11.56  (9.18 – 15.44) | 17.60  (10.10 – 22.36) | 0.267 |  | IL-7, $\mu$g/mL  median (IQR) | 0.09  (0.03 – 0.29) | 0.13  (0.09 – 0.29) | 0.430 |
| IL-8, pg/mL  median (IQR) |  | 9.50  (5.54 – 14.10) | 9.42  (6.22 – 11.84) | 0.892 |  | IL-8, $\mu$g/mL  median (IQR) | 16.98  (2.60 – 34.32) | 52.82  (26.75 – 134.70) | 0.075 |
| IL-10, pg/mL  median (IQR) |  | 28.52  (28.52 – 39.02) | 33.78  (23.12 – 49.38) | 0.454 |  | IL-10, $\mu$g/mL  median (IQR) | 1.07  (0.38 – 4.13) | 1.24  (1.13 – 6.62) | 0.443 |
| IL-12 p70, pg/mL  median (IQR) |  | - | - | - |  | IL-12p70, $\mu$g/mL  median (IQR) | 0.07  (0.02 – 0.23) | 0.12  (0.06 – 0.37) | 0.336 |
| IL-13, pg/mL  median (IQR) |  | - | - | - |  | IL-13, $\mu$g/mL  median (IQR) | 0.43  (0.05 – 1.42) | 0.45  (0.35 – 1.45) | 0.566 |
| IL-15, pg/mL  median (IQR) |  | 1.78  (1.78 – 2.56) | 1.56  (0.94 – 3.00) | 0.560 |  | IL-15, $\mu$g/mL  median (IQR) | 0.04  (0.01 – 0.12) | 0.05  (0.04 – 0.16) | 0.430 |
| IL-17, pg/mL  median (IQR) |  | - | - | - |  | IL-17, $\mu$g/mL  median (IQR) | 0.04  (0.01 – 0.11) | 0.04  (0.03 – 0.15) | 0.687 |
| IL-25, pg/mL  median (IQR) |  | - | - | - |  | IL-25, $\mu$g/mL  median (IQR) | 0.09  (0.06 – 0.79) | 0.18  (0.14 – 0.93) | 0.387 |
| IL-33, pg/mL  median (IQR) |  | 6.12  (4.24 – 7.78) | 7.78  (4.24 – 16.48) | 0.647 |  | IL-33, $\mu$g/mL  median (IQR) | 0.51  (0.19 – 1.81) | 0.79  (0.57 – 2.04) | 0.633 |
| ECP, kU/L  median (IQR) |  | 6.70  (3.61 – 12.13) | 5.77  (1.89 – 20.96) | 0.743 |  | ECP, g/L  median (IQR) | 0.11  (0.03 – 0.24) | 0.29  (0.02 – 0.62) | 0.566 |
| MPO, ng/mL  median (IQR) |  | 164.23  (129.84 – 579.62) | 199.55  (181.70 – 260.61) | 0.743 |  | MPO, mg/mL  median (IQR) | 0.02  (0.01 – 0.08) | 0.03  (0.02 – 0.08) | 0.519 |
| Total IgE, kU/L  median (IQR) |  | 139.00  (69.30 – 280.00) | 120.00  (90.90 – 303.50) | 0.913 |  | Total IgE, kU/L  median (IQR) | 50.08  (19.75 – 161.12) | 90.72  (44.07 – 103.74) | 0.849 |
| Periostin, ng/mL  median (IQR) |  | 240.86  (217.22 – 262.42) | 266.78  (202.75 – 271.65) | 0.441 |  | Periostin, mg/mL  median (IQR) | 2.22  (0.72 – 9.57) | 4.07  (2.03 – 6.97) | 0.566 |
| CD40L, pg/mL  median (IQR) |  | 164.96  (164.96 – 2225.78) | 164.96  (164.96 -707.20) | 1.000 |  | CD40L, $\mu$g/mL  median (IQR) | 23.11  (3.43 – 77.57) | 30.51  (19.13 – 91.91) | 0.503 |
| EGF, pg/mL  median (IQR) |  | 32.88  (3.04 – 192.60) | 20.04  (3.04 – 64.96) | 0.432 |  | EGF, $\mu$g/mL  median (IQR) | 2.56  (1.22 – 11.15) | 5.87  (2.05 – 8.07) | 0.849 |
| Eotaxin, pg/mL  median (IQR) |  | 130.44  (106.66 – 222.02) | 116.48  (85.34 – 170.82) | 0.390 |  | Eotaxin, $\mu$g/mL  median (IQR) | 1.54  (0.39 – 4.75) | 1.84  (1.22 – 5.56) | 0.503 |
| FGF-basic, pg/mL  median (IQR) |  | 3.42  (0.64 – 5.20) | 3.42  (0.64 – 5.24) | 1.000 |  | FGF-basic, $\mu$g/mL  median (IQR) | 0.35  (0.08 – 0.90) | 0.32  (0.22 – 1.08) | 0.703 |
| Flt-3 Ligand, pg/mL  median (IQR) |  | 89.12  (76.62 – 98.86) | 85.86  (67.30 – 126.26) | 0.762 |  | Flt-3 Ligand, $\mu$g/mL  median (IQR) | 0.85  (0.19 – 1.99) | 1.25  (0.86 – 3.52) | 0.208 |
| G-CSF, pg/mL  median (IQR) |  | 66.74  (45.14 – 83.60) | 85.58  (40.44 – 95.62) | 0.875 |  | G-CSF, $\mu$g/mL  median (IQR) | 1.70  (1.00 – 7.11) | 12.74  (4.83 – 81.81) | 0.075 |
| GM-CSF, pg/mL  median (IQR) |  | 17.40  (15.79 – 25.84) | 22.48  (19.1 – 25.84) | 0.391 |  | GM-CSF, $\mu$g/mL  median (IQR) | 0.60  (0.22 – 1.80) | 0.78  (0.60 – 3.76) | 0.289 |
| Granzyme B, pg/mL  median (IQR) |  | - | - | - |  | Granzyme B, $\mu$g/mL  median (IQR) | 0.93  (0.27 – 2.56 | 1.16  (0.56 – 5.66) | 0.703 |
| CXCL-1, pg/mL  median (IQR) |  | 91.86  (60.00 – 138.46) | 104.96  (73.40 – 186.22) | 0.393 |  | CXCL-1, $\mu$g/mL  median (IQR) | 60.28  (28.79 – 203.19) | 126.90  (58.91 – 376.12) | 0.289 |
| CXCL-2, pg/mL  median (IQR) |  | 515.47  (408.64 – 883.04) | 637.48  (359.36 – 1716.06) | 0.441 |  | CXCL-2, $\mu$g/mL  median (IQR) | 2.00  (1.00 – 4.70) | 6.51  (2.84 – 10.92) | 0.117 |
| IFN-α , pg/mL  median (IQR) |  | - | - | - |  | IFN-α, $\mu$g/mL  median (IQR) | 0.13  (0.03 – 0.56) | 0.12  (0.11 – 0.50) | 1.000 |
| IFN-γ, pg/mL  median (IQR) |  | - | - | - |  | IFN-γ, $\mu$g/mL  median (IQR) | 0.25  (0.04 – 0.67) | 0.31  (0.18 – 0.88) | 0.503 |
| IP-10, pg/mL  median (IQR) |  | 91.86  (59.98 – 138.46) | 104.96  (73.40 – 186.22) | 0.393 |  | IP-10, $\mu$g/mL  median (IQR) | 2.38  (1.15 – 5.53) | 3.36  (1.36 – 46.88) | 0.633 |
| CCL-2, pg/mL  median (IQR) |  | 248.12  (199.26 – 336.60) | 284.18  (146.08 – 345.20) | 1.000 |  | CCL-2, $\mu$g/mL  median (IQR) | 0.31  (0.57 – 2.84) | 1.94  (1.32 – 2.42) | 1.000 |
| CCL-3, pg/mL  median (IQR) |  | 106.62  (89.10 – 434.92) | 75.50  (70.92 – 91.86) | **0.036*** |  | CCL-3, $\mu$g/mL  median (IQR) | 0.43  (0.11 – 1.42) | 0.93  (0.23 – 1.87) | 0.503 |
| CCL-4, pg/mL  median (IQR) |  | 1019.02  (915.42 – 2772.02) | 841.06  (617.66 – 907.50) | **0.009**** |  | CCL-4, $\mu$g/mL  median (IQR) | 4.83  (1.14 – 12.90) | 8.78  (3.41 – 13.72) | 0.516 |
| CCL-5, ng/mL  median (IQR) |  | 38.04  (29.96 – 53.10) | 59.48  (20.62 – 82.32) | 0.441 |  | CCL-5, $m$g/mL  median (IQR) | 1.88  (0.31 – 6.43) | 1.89  (0.69 – 5.78) | 0.703 |
| CCL-19, pg/mL  median (IQR) |  | 84.90  (61.48 – 92.80) | 110.68  (73.76 – 177.70) | 0.441 |  | CCL-19, $\mu$g/mL  median (IQR) | 1.62  (0.66 – 4.06) | 4.77  (2.05 – 9.29) | 0.123 |
| CCL-20, pg/mL  median (IQR) |  | 10.40  (3.90 – 18.56) | 12.20  (6.26 – 14.26) | 0.980 |  | CCL-20, $\mu$g/mL  median (IQR) | 0.38  (0.10 – 0.57) | 0.76  (0.35 – 4.13) | 0.117 |
| PD-L1, pg/mL  median (IQR) |  | 29.46  (24.30 – 61.00) | 25.40  (19.18 – 52.94) | 0.893 |  | PD-L1, $\mu$g/mL  median (IQR) | 1.30  (0.37 – 4.97) | 1.45  (0.97 – 4.66) | 0.566 |
| PDGF-AA, pg/mL  median (IQR) |  | - | - | - |  | PDGF-AA, $\mu$g/mL  median (IQR) | 2.37  (0.67 – 5.23) | 2.94  (1.87 – 3.29) | 1.000 |
| PDGF-AB, pg/mL  median (IQR) |  | 3015.32  (1699.74 – 4126.84) | 2843.92  (1691.60 – 3921.40) | 0.661 |  | PDGF-AB, $\mu$g/mL  median (IQR) | 0.05  (0.01 – 0.11) | 0.09  (0.04 – 0.24) | 0.289 |
| TGF-α, pg/mL  median (IQR) |  | 8.50  (3.90 – 11.54) | 8.50  (2.36 – 17.66) | 1.000 |  | TGF-α, $\mu$g/mL  median (IQR) | 0.59  (0.12 – 1.45) | 0.48  (0.38 – 1.43) | 0.703 |
| TNF-α, pg/mL  median (IQR) |  | 4.02  (0.94 – 4.80) | 4.04  (1.70 – 5.96) | 0.804 |  | TNF-α, $\mu$g/mL  median (IQR) | 0.17  (0.05 – 0.30) | 0.53  (0.17 – 1.19) | 0.173 |
| TRAIL, pg/mL  median (IQR) |  | 33.86  (18.62 – 55.54) | 44.70  (21.88 – 49.04) | 0.848 |  | TRAIL, $\mu$g/mL  median (IQR) | 6.42  (2.66 – 26.10) | 8.39  (5.60 – 31.94) | 0.566 |
| VEGF, pg/mL  median (IQR) |  | 117.94  (81.74 – 163.42) | 114.40  (107.18 – 239.54) | 0.762 |  | VEGF, $\mu$g/mL  median (IQR) | 2.11  (1.17 – 6.21) | 2.70  (2.35 – 5.75) | 0.703 |

*, *P* < 0.05; **, *P* < 0.01.

**Table S3.** The difference of biomarkers based on the nasal congestion score (NCS) improvement.

| Characteristics |  | Serum (N = 16) | | |  | Characteristics | Nasal secretion (N = 18) | | |
| --- | --- | --- | --- | --- | --- | --- | --- | --- | --- |
|  |  | Yes | No | *P* value |  |  | Yes | No | *P* value |
| IL-1α, pg/mL  median (IQR) |  | 15.76  (10.00 – 17.48) | 15.04  (11.26 – 16.86) | 0.732 |  | IL-1α, $\mu$g/mL  median (IQR) | 0.34  (0.06 – 0.83) | 0.24  (0.11 –0.92) | 0.838 |
| IL-1β, pg/mL  median (IQR) |  | 5.52  (2.74 – 6.34) | 5.82  (3.38 – 7.88) | 0.507 |  | IL-1β, $\mu$g/mL  median (IQR) | 0.15  (0.02 – 0.45) | 0.52  (0.10 – 0.80) | 0.424 |
| IL-1 ra, pg/mL  median (IQR) |  | 401.40  (311.46 – 492.96) | 394.84  (357.70 – 474.56) | 1.000 |  | IL-1 ra, $\mu$g/mL  median (IQR) | 78.32  (21.72 – 349.86) | 69.78  (44.74 – 144.52) | 0.946 |
| IL-2, pg/mL  median (IQR) |  | - | - | - |  | IL-2, $\mu$g/mL  median (IQR) | 0.17  (0.03 – 0.42) | 0.12  (0.09 – 0.37) | 0.892 |
| IL-3, pg/mL  median (IQR) |  | - | - | - |  | IL-3, $\mu$g/mL  median (IQR) | 0.53  (0.06 – 1.21) | 0.24  (0.17 – 0.66) | 0.682 |
| IL-4, pg/mL  median (IQR) |  | - | - | - |  | IL-4, $\mu$g/mL  median (IQR) | 0.03  (0.00 – 0.08) | 0.02  (0.01 – 0.13) | 0.820 |
| IL-5, pg/mL  median (IQR) |  | - | - | - |  | IL-5, $\mu$g/mL  median (IQR) | 0.09  (0.02 – 0.11) | 0.07  (0.04 – 0.24) | 0.945 |
| IL-6, pg/mL  median (IQR) |  | 13.62  (6.06 – 16.18) | 9.40  (6.06 – 17.12) | 0.570 |  | IL-6, $\mu$g/mL  median (IQR) | 0.72  (0.22 – 1.66) | 0.44  (0.22 – 1.17) | 0.385 |
| IL-7, pg/mL  median (IQR) |  | 11.22  (8.14 – 15.22) | 16.20  (11.60 – 18.86) | 0.147 |  | IL-7, $\mu$g/mL  median (IQR) | 0.11  (0.04 – 0.30) | 0.12  (0.06 – 0.18) | 0.982 |
| IL-8, pg/mL  median (IQR) |  | 6.82  (4.86 – 9.56) | 12.40  (9.88 – 20.36) | **0.010*** |  | IL-8, $\mu$g/mL  median (IQR) | 19.21  (4.09 – 85.84) | 32.86  (12.12 – 67.22) | 0.682 |
| IL-10, pg/mL  median (IQR) |  | 31.14  (28.52 – 39.66) | 31.14  (16.52 – 44.20) | 0.730 |  | IL-10, $\mu$g/mL  median (IQR) | 1.30  (0.52 – 5.74) | 1.24  (0.83 – 3.15) | 0.964 |
| IL-12 p70, pg/mL  median (IQR) |  | - | - | - |  | IL-12p70, $\mu$g/mL  median (IQR) | 0.09  (0.02 – 0.30) | 0.07  (0.05 – 0.36) | 1.000 |
| IL-13, pg/mL  median (IQR) |  | - | - | - |  | IL-13, $\mu$g/mL  median (IQR) | 0.66  (0.06 – 1.48) | 0.40  (0.21 – 1.58) | 0.750 |
| IL-15, pg/mL  median (IQR) |  | 1.78  (1.44 – 2.44) | 2.16  (1.38 – 2.66) | 0.939 |  | IL-15, $\mu$g/mL  median (IQR) | 0.07  (0.01 – 0.16) | 0.04  (0.03 – 0.12) | 0.801 |
| IL-17, pg/mL  median (IQR) |  | - | - | - |  | IL-17, $\mu$g/mL  median (IQR) | 0.07  (0.01 – 0.11) | 0.04  (0.02 – 0.23) | 0.838 |
| IL-25, pg/mL  median (IQR) |  | - | - | - |  | IL-25, $\mu$g/mL  median (IQR) | 0.44  (0.06 – 0.83) | 0.14  (0.07 – 1.30) | 0.820 |
| IL-33, pg/mL  median (IQR) |  | 5.28  (4.24 – 7.78) | 6.96  (3.42 – 13.36) | 0.656 |  | IL-33, $\mu$g/mL  median (IQR) | 0.72  (0.15 – 1.93) | 0.83  (0.63 – 1.29) | 0.750 |
| ECP, kU/L  median (IQR) |  | 5.47  (3.35 – 10.62) | 7.56  (4.06 – 18.71) | 0.428 |  | ECP, g/L  median (IQR) | 0.12  (0.02 – 0.47) | 0.14  (0.01 – 0.39) | 0.964 |
| MPO, ng/mL  median (IQR) |  | 188.89  (131.58 – 347.64) | 238.62  (166.06 – 431.95) | 0.562 |  | MPO, mg/mL  median (IQR) | 0.03  (0.01 – 0.10) | 0.03  (0.01 – 0.05) | 0.801 |
| Total IgE, kU/L  median (IQR) |  | 145.50  (67.78 – 284.25) | 129.50  (120.00 – 139.00) | 0.875 |  | Total IgE, kU/L  median (IQR) | 70.34  (25.98 – 163.91) | 75.57  (20.96 – 107.46) | 0.682 |
| Periostin, ng/mL  median (IQR) |  | 235.83  (204.52 – 259.75) | 264.60  (240.57 – 271.39) | 0.118 |  | Periostin, mg/mL  median (IQR) | 2361.02  (852.76 – 8863.94) | 2848.74  (1324.54 – 6143.99) | 1.000 |
| CD40L, pg/mL  median (IQR) |  | 164.96  (164.96 – 2065.14) | 164.96  (164.96 – 1365.24) | 1.000 |  | CD40L, $\mu$g/mL  median (IQR) | 25.09  (5.03 – 84.15) | 65.56  (19.13 – 112.00) | 0.820 |
| EGF, pg/mL  median (IQR) |  | 31.50  (3.04 – 177.38) | 28.50  (15.22 – 154.10) | 0.872 |  | EGF, $\mu$g/mL  median (IQR) | 4.47  (1.71 – 9.82) | 2.32  (1.57 – 9.52) | 0.616 |
| Eotaxin, pg/mL  median (IQR) |  | 128.16  (103.18 – 195.62) | 139.74  (85.34 – 179.74) | 0.731 |  | Eotaxin, $\mu$g/mL  median (IQR) | 1.91  (0.61 – 4.97) | 1.41  (0.92 – 3.33) | 0.750 |
| FGF-basic, pg/mL  median (IQR) |  | 3.42  (0.64 – 3.86) | 3.42  (0.64 – 8.72) | 1.000 |  | FGF-basic, $\mu$g/mL  median (IQR) | 0.40  (0.09 – 0.98) | 0.27  (0.17 – 0.82) | 0.750 |
| Flt-3 Ligand, pg/mL  median (IQR) |  | 83.96  (59.88 – 106.28) | 94.54  (82.86 – 1062.38) | 0.326 |  | Flt-3 Ligand, $\mu$g/mL  median (IQR) | 0.89  (0.29 – 2.53) | 1.16  (0.51 – 1.81) | 0.892 |
| G-CSF, pg/mL  median (IQR) |  | 71.94  (41.86 – 96.78) | 67.16  (52.26 – 87.94) | 0.326 |  | G-CSF, $\mu$g/mL  median (IQR) | 3.69  (1.08 – 11.76) | 1.32  (1.01 – 1.63) | 0.820 |
| GM-CSF, pg/mL  median (IQR) |  | 21.64  (14.84 – 25.84) | 19.94  (16.98 – 25.84) | 0.936 |  | GM-CSF, $\mu$g/mL  median (IQR) | 0.62  (0.31 – 3.21) | 0.77  (0.36 – 1.29) | 0.892 |
| Granzyme B, pg/mL  median (IQR) |  | - | - | - |  | Granzyme B, $\mu$g/mL  median (IQR) | 1.26  (0.34 – 7.62) | 1.03  (0.23 – 1.38) | 0.437 |
| CXCL-1, pg/mL  median (IQR) |  | 119.60  (80.38 – 162.08) | 93.96  (36.24 –111.62) | 0.208 |  | CXCL-1, $\mu$g/mL  median (IQR) | 70.25  (40.83 – 252.44) | 97.56  (40.80 – 186.26) | 1.000 |
| CXCL-2, pg/mL  median (IQR) |  | 508.88  (362.90 – 993.02) | 581.18  (440.26 – 989.82) | 0.562 |  | CXCL-2, $\mu$g/mL  median (IQR) | 2.74  (1.46 – 8.91) | 4.04  (1.96 – 7.05) | 0.682 |
| IFN-α, pg/mL  median (IQR) |  | - | - | - |  | IFN-α, $\mu$g/mL  median (IQR) | 0.18  (0.03 – 0.53) | 0.10  (0.02 – 0.38) | 0.616 |
| IFN-γ, pg/mL  median (IQR) |  | - | - | - |  | IFN-γ, $\mu$g/mL  median (IQR) | 0.26  (0.09 – 0.70) | 0.24  (0.11 – 0.56) | 0.892 |
| IP-10, pg/mL  median (IQR) |  | 119.60  (80.38 – 162.08) | 93.96  (36.24 – 111.62) | 0.208 |  | IP-10, $\mu$g/mL  median (IQR) | 3.61  (1.56 – 32.82) | 1.80  (1.23 – 3.83) | 0.250 |
| CCL-2, pg/mL  median (IQR) |  | 247.44  (195.20 – 329.12) | 288.32  (199.02 – 343.86) | 0.635 |  | CCL-2, $\mu$g/mL  median (IQR) | 2.20  (0.86 – 2.62) | 1.94  (0.90 – 2.51) | 0.964 |
| CCL-3, pg/mL  median (IQR) |  | 98.18  (73.20 – 457.02) | 89.72  (80.92 – 105.74) | 0.827 |  | CCL-3, $\mu$g/mL  median (IQR) | 0.70  (0.22 – 1.44) | 0.37  (0.13 – 2.27) | 0.964 |
| CCL-4, pg/mL  median (IQR) |  | 1060.82  (862.78 –3154.90) | 898.18  (817.28 – 955.28) | 0.181 |  | CCL-4, $\mu$g/mL  median (IQR) | 5.02  (2.23 – 13.33) | 4.45  (2.19 – 16.32) | 0.945 |
| CCL-5, ng/mL  median (IQR) |  | 34.94  (25.90 – 55.10) | 33.94  (19.84 – 48.02) | 0.492 |  | CCL-5, $m$g/mL  median (IQR) | 2.14  (0.40 – 6.54) | 20.47  (1.89 – 39.05) | 0.682 |
| CCL-19, pg/mL  median (IQR) |  | 87.32  (63.78 – 215.66) | 91.20  (52.66 – 113.18) | 0.792 |  | CCL-19, $\mu$g/mL  median (IQR) | 1.92  (0.93 – 9.90) | 1.70  (0.43 – 4.77) | 0.892 |
| CCL-20, pg/mL  median (IQR) |  | 11.30  (3.90 – 18.76) | 11.30  (6.34 – 13.82) | 0.771 |  | CCL-20, $\mu$g/mL  median (IQR) | 0.39  (0.13 – 0.73) | 0.44  (0.17 – 1.11) | 0.909 |
| PD-L1, pg/mL  median (IQR) |  | 24.86  (21.46 – 55.88) | 29.46  (22.28 – 64.86) | 0.617 |  | PD-L1, $\mu$g/mL  median (IQR) | 1.53  (0.58 – 4.98) | 1.27  (0.68 – 3.77) | 0.964 |
| PDGF-AA, pg/mL  median (IQR) |  | - | - | - |  | PDGF-AA, $\mu$g/mL  median (IQR) | 2.66  (0.74 – 4.46) | 2.83  (1.08 – 5.39) | 0.682 |
| PDGF-AB, pg/mL  median (IQR) |  | 3097.64  (1666.20 – 4455.30) | 2875.00  (2262.14 – 3865.84) | 0.792 |  | PDGF-AB, $\mu$g/mL  median (IQR) | 0.06  (0.02 – 0.15) | 0.05  (0.03 – 0.14) | 0.750 |
| TGF-α, pg/mL  median (IQR) |  | 9.26  (3.52 – 13.46) | 8.10  (5.82 – 13.84) | 1.000 |  | TGF-α, $\mu$g/mL  median (IQR) | 0.61  (0.12 – 1.49) | 0.45  (0.27 – 1.32) | 0.892 |
| TNF-α, pg/mL  median (IQR) |  | 3.26  (0.94 – 4.24) | 4.80  (3.26 – 9.04) | 0.088 |  | TNF-α, $\mu$g/mL  median (IQR) | 0.20  (0.09 – 0.48) | 0.21  (0.07 – 0.69) | 0.964 |
| TRAIL, pg/mL  median (IQR) |  | 38.28  (22.96 – 57.18) | 31.68  (18.06 – 42.54) | 0.474 |  | TRAIL, $\mu$g/mL  median (IQR) | 9.39  (3.76 – 32.52) | 5.99  (4.16 – 15.02) | 0.616 |
| VEGF, pg/mL  median (IQR) |  | 110.12  (78.14 – 141.02) | 124.74  (110.12 – 230.54) | 0.171 |  | VEGF, $\mu$g/mL  median (IQR) | 3.21  (1.39 – 6.94) | 2.66  (1.83 – 4.81) | 0.892 |

*, *P* < 0.05.

**Table S4.** The difference of biomarkers based on the runny nose score (RNS) improvement.

| Characteristics |  | Serum (N = 16) | | |  | Characteristics | Nasal secretion (N = 18) | | |
| --- | --- | --- | --- | --- | --- | --- | --- | --- | --- |
|  |  | Yes | No | *P* value |  |  | Yes | No | *P* value |
| IL-1α, pg/mL  median (IQR) |  | 15.52  (12.62 – 17.58) | 7.16  (3.08 – 15.52) | 0.079 |  | IL-1α, $\mu$g/mL  median (IQR) | 0.31  (0.05 – 0.79) | 0.25  (0.13 – 0.97) | 0.811 |
| IL-1β, pg/mL  median (IQR) |  | 5.72  (3.60 – 7.38) | 2.74  (1.40 – 6.14) | 0.259 |  | IL-1β, $\mu$g/mL  median (IQR) | 0.24  (0.02 – 0.66) | 0.13  (0.02 – 0.42) | 0.510 |
| IL-1 ra, pg/mL  median (IQR) |  | 413.60  (327.06 – 501.82) | 376.08  (319.50 – 464.08) | 0.800 |  | IL-1 ra, $\mu$g/mL  median (IQR) | 55.32  (20.41 – 340.58) | 79.17  (60.38 – 205.65) | 0.667 |
| IL-2, pg/mL  median (IQR) |  | - | - | - |  | IL-2, $\mu$g/mL  median (IQR) | 0.13  (0.03 – 0.39) | 0.14  (0.11 – 0.43) | 0.738 |
| IL-3, pg/mL  median (IQR) |  | - | - | - |  | IL-3, $\mu$g/mL  median (IQR) | 0.32  (0.06 – 1.24) | 0.33  (0.21 – 0.74) | 1.000 |
| IL-4, pg/mL  median (IQR) |  | - | - | - |  | IL-4, $\mu$g/mL  median (IQR) | 0.02  (0.00 – 0.06) | 0.03  (0.01 – 0.16) | 0.574 |
| IL-5, pg/mL  median (IQR) |  | - | - | - |  | IL-5, $\mu$g/mL  median (IQR) | 0.08  (0.02 – 0.10) | 0.14  (0.05 – 0.17) | 0.293 |
| IL-6, pg/mL  median (IQR) |  | 13.62  (8.30 – 16.70) | 2.64  (2.64 – 13.62) | 0.141 |  | IL-6, $\mu$g/mL  median (IQR) | 0.61  (0.14 – 1.68) | 0.53  (0.40 – 1.57) | 1.000 |
| IL-7, pg/mL  median (IQR) |  | 13.24  (100.02 – 17.28) | 11.72  (8.48 – 22.60) | 1.000 |  | IL-7, $\mu$g/mL  median (IQR) | 0.12  (0.03 – 0.31) | 0.11  (0.07 – 0.23) | 0.985 |
| IL-8, pg/mL  median (IQR) |  | 9.50  (5.54 – 12.78) | 8.54  (3.92 – 13.34) | 0.687 |  | IL-8, $\mu$g/mL  median (IQR) | 20.79  (2.60 – 101.72) | 38.21  (15.30 – 52.82) | 0.738 |
| IL-10, pg/mL  median (IQR) |  | 28.52  (28.52 – 402.32) | 33.78  (12.58 – 33.78) | 0.570 |  | IL-10, $\mu$g/mL  median (IQR) | 1.24  (0.50 – 5.36) | 1.24  (1.03 – 5.87) | 0.912 |
| IL-12 p70, pg/mL  median (IQR) |  | - | - | - |  | IL-12p70, $\mu$g/mL  median (IQR) | 0.07  (0.02 – 0.24) | 0.12  (0.06 – 0.32) | 0.574 |
| IL-13, pg/mL  median (IQR) |  | - | - | - |  | IL-13, $\mu$g/mL  median (IQR) | 0.43  (0.05 – 1.29) | 0.45  (0.43 – 1.87) | 0.498 |
| IL-15, pg/mL  median (IQR) |  | 1.78  (1.66 – 2.60) | 1.00  (0.90 – 2.66) | 0.257 |  | IL-15, $\mu$g/mL  median (IQR) | 0.15  (0.05 – 0.35) | 0.16  (0.05 – 0.16) | 0.901 |
| IL-17, pg/mL  median (IQR) |  | - | - | - |  | IL-17, $\mu$g/mL  median (IQR) | 0.41  (0.01 – 0.11) | 0.04  (0.04 – 0.21) | 0.488 |
| IL-25, pg/mL  median (IQR) |  | - | - | - |  | IL-25, $\mu$g/mL  median (IQR) | 0.13  (0.06 – 0.71) | 0.18  (0.14 – 1.15) | 0.498 |
| IL-33, pg/mL  median (IQR) |  | 6.12  (4.34 – 8.60) | 4.24  (4.24 – 7.78) | 0.434 |  | IL-33, $\mu$g/mL  median (IQR) | 0.85  (0.26 – 2.05) | 0.69  (0.46 – 0.96) | 0.824 |
| ECP, kU/L  median (IQR) |  | 6.70  (3.50 – 12.74) | 5.77  (0.39 – 7.11) | 0.439 |  | ECP, g/L  median (IQR) | 0.12  (0.03 – 0.55) | 0.02  (0.01 – 0.29) | 0.426 |
| MPO, ng/mL  median (IQR) |  | 229.23  (131.16 – 481.17) | 185.27  (178.13 – 199.55) | 0.900 |  | MPO, mg/mL  median (IQR) | 0.02  (0.01 – 0.10) | 0.03  (0.02 – 0.06) | 0.938 |
| Total IgE, kU/L  median (IQR) |  | 123.00  (73.45 – 239.50) | 168.00  (120.00 – 439.00) | 0.364 |  | Total IgE, kU/L  median (IQR) | 50.08  (22.39 – 149.19) | 93.56  (64.74 – 113.93) | 0.574 |
| Periostin, ng/mL  median (IQR) |  | 245.21  (219.76 – 264.60) | 271.13  (160.29 – 272.17) | 0.611 |  | Periostin, mg/mL  median (IQR) | 2.45  (7.43 – 8.96) | 4.07  (1.60 – 5.36) | 0.824 |
| CD40L, pg/mL  median (IQR) |  | 164.96  (164.96 – 2118.68) | 164.96  (164.96 –164.96) | 0.250 |  | CD40L, $\mu$g/mL  median (IQR) | 23.11  (4.00 – 84.91) | 30.51  (19.13 – 81.87) | 0.738 |
| EGF, pg/mL  median (IQR) |  | 36.94  (11.16 – 182.46) | 3.04  (3.04 – 20.04) | 0.109 |  | EGF, $\mu$g/mL  median (IQR) | 3.07  (1.48 – 10.66) | 2.33  (1.76 – 7.33) | 0.738 |
| Eotaxin, pg/mL  median (IQR) |  | 130.44  (99.70 – 200.34) | 116.48  (85.34 – 162.98) | 0.454 |  | Eotaxin, $\mu$g/mL  median (IQR) | 1.54  (0.56 – 5.19) | 1.84  (1.16 – 3.67) | 0.912 |
| FGF-basic, pg/mL  median (IQR) |  | 3.42  (0.64 – 6.14) | 3.42  (0.64 – 3.42) | 0.529 |  | FGF-basic, $\mu$g/mL  median (IQR) | 0.35  (0.09 – 0.99) | 0.32  (0.21 – 0.93) | 1.000 |
| Flt-3 Ligand, pg/mL  median (IQR) |  | 89.12  (79.34 – 101.82) | 73.88  (60.72 – 128.92) | 0.782 |  | Flt-3 Ligand, $\mu$g/mL  median (IQR) | 0.93  (0.24 – 2.14) | 1.25  (0.63 – 2.66) | 0.574 |
| G-CSF, pg/mL  median (IQR) |  | 68.00  (51.00 – 95.62) | 42.34  (38.54 – 85.58) | 0.364 |  | G-CSF, $\mu$g/mL  median (IQR) | 3.40  (1.03 – 8.66) | 12.74  (1.01 – 34.41) | 0.498 |
| GM-CSF, pg/mL  median (IQR) |  | 22.48  (15.70 – 23.84) | 20.80  (17.40 – 22.48) | 0.839 |  | GM-CSF, $\mu$g/mL  median (IQR) | 0.65  (0.28 – 2.36) | 0.76  (0.44 – 2.92) | 0.824 |
| Granzyme B, pg/mL  median (IQR) |  | - | - | - |  | Granzyme B, $\mu$g/mL  median (IQR) | 0.94  (0.24 – 3.53) | 1.16  (0.89 – 2.35) | 0.738 |
| CXCL-1, pg/mL  median (IQR) |  | 100.74  (73.58 – 143.40) | 100.74  (46.08 – 224.14) | 0.821 |  | CXCL-1, $\mu$g/mL  median (IQR) | 68.23  (40.49 – 237.05) | 126.90  (49.59 – 220.14) | 0.738 |
| CXCL-2, pg/mL  median (IQR) |  | 515.46  (210.20 – 932.02) | 637.48  (214.68 – 2121.92) | 0.704 |  | CXCL-2, $\mu$g/mL  median (IQR) | 3.30  (1.35 – 4.79) | 6.51  (2.39 – 10.35) | 0.301 |
| IFN-α, pg/mL  median (IQR) |  | - | - | - |  | IFN-α, $\mu$g/mL  median (IQR) | 0.13  (0.0.3 – 0.53) | 0.12  (0.12 – 0.53) | 1.000 |
| IFN-γ, pg/mL  median (IQR) |  | - | - | - |  | IFN-γ, $\mu$g/mL  median (IQR) | 0.25  (0.05 – 0.70) | 0.31  (0.13 – 0.68) | 0.824 |
| IP-10, pg/mL  median (IQR) |  | 100.74  (73.58 – 143.40) | 100.74  (46.08 – 224.14) | 0.821 |  | IP-10, $\mu$g/mL  median (IQR) | 2.68  (1.39 – 5.81) | 1.38  (1.34 – 38.75) | 0.738 |
| CCL-2, pg/mL  median (IQR) |  | 251.86  (217.60 – 330.70) | 201.88  (90.26 – 365.64) | 0.521 |  | CCL-2, $\mu$g/mL  median (IQR) | 2.33  (0.82 – 2.66) | 1.54  (1.09 – 1.94) | 0.426 |
| CCL-3, pg/mL  median (IQR) |  | 90.34  (84.36 – 280.20) | 75.50  (75.50 – 101.00) | 0.287 |  | CCL-3, $\mu$g/mL  median (IQR) | 0.47  (0.17 – 1.61) | 0.31  (0.16 – 0.93) | 0.498 |
| CCL-4, pg/mL  median (IQR) |  | 998.40  (886.76 – 2024.38) | 841.06  (493.42 – 934.04) | 0.146 |  | CCL-4, $\mu$g/mL  median (IQR) | 4.92  (1.72 – 13.75) | 4.07  (2.74 – 8.78) | 0.725 |
| CCL-5, ng/mL  median (IQR) |  | 48.02  (30.58 – 55.90) | 21.38  (19.84 – 71.26) | 0.521 |  | CCL-5, $m$g/mL  median (IQR) | 1.88  (0.36 – 6.21) | 1.89  (0.81 – 9.11) | 0.654 |
| CCL-19, pg/mL  median (IQR) |  | 89.76  (63.02 – 106.74) | 110.68  (115.56 – 146.70) | 0.704 |  | CCL-19, $\mu$g/mL  median (IQR) | 1.62  (0.88 – 5.67) | 2.31  (1.79 – 10.52) | 0.426 |
| CCL-20, pg/mL  median (IQR) |  | 10.40  (5.24 – 18.26) | 12.20  (0.34 – 13.40) | 0.627 |  | CCL-20, $\mu$g/mL  median (IQR) | 0.38  (0.11 – 0.91) | 0.52  (0.19 – 0.76) | 0.841 |
| PD-L1, pg/mL  median (IQR) |  | 129.46  (24.30 – 68.70) | 25.40  (12.94 – 29.46) | 0.464 |  | PD-L1, $\mu$g/mL  median (IQR) | 1.30  (0.53 – 4.98) | 1.45  (0.84 – 3.74) | 1.000 |
| PDGF-AA, pg/mL  median (IQR) |  | - | - | - |  | PDGF-AA, $\mu$g/mL  median (IQR) | 2.56  (0.78 – 5.09) | 3.09  (1.18 – 3.50) | 1.000 |
| PDGF-AB, pg/mL  median (IQR) |  | 3179.94  (2107.72 – 4095.40) | 1881.76  (1501.46 – 2843.92) | 0.146 |  | PDGF-AB, $\mu$g/mL  median (IQR) | 0.06  (0.02 – 0.11) | 0.09  (0.03 – 0.17) | 0.654 |
| TGF-α, pg/mL  median (IQR) |  | 10.02  (5.42 – 13.84) | 3.66  (3.66 – 8.50) | 0.095 |  | TGF-α, $\mu$g/mL  median (IQR) | 0.59  (0.12 – 1.31) | 0.48  (0.43 – 1.55) | 0.738 |
| TNF-α, pg/mL  median (IQR) |  | 4.02  (1.32 – 5.96) | 4.04  (0.94 – 4.80) | 0.945 |  | TNF-α, $\mu$g/mL  median (IQR) | 0.17  (0.09 – 0.32) | 0.53  (0.09 – 0.56) | 0.654 |
| TRAIL, pg/mL  median (IQR) |  | 36.02  (21.88– 52.30) | 27.32  (16.42 – 44.70) | 0.455 |  | TRAIL, $\mu$g/mL  median (IQR) | 8.14  (3.54 – 33.10) | 6.24  (4.96 – 30.79) | 1.000 |
| VEGF, pg/mL  median (IQR) |  | 117.94  (89.24 – 164.90) | 114.40  (112.04 – 117.94) | 0.920 |  | VEGF, $\mu$g/mL  median (IQR) | 2.70  (1.20 – 6.25) | 2.63  (2.07 – 4.31) | 0.824 |

**Table S5.** The difference of biomarkers based on the sense of smell score (SSS) improvement.

| Characteristics |  | Serum (N = 16) | | |  | Characteristics | Nasal secretion (N = 18) | | |
| --- | --- | --- | --- | --- | --- | --- | --- | --- | --- |
|  |  | Yes | No | *P* value |  |  | Yes | No | *P* value |
| IL-1α, pg/mL  median (IQR) |  | 9.76  (9.42 – 17.14) | 14.58  (7.16 – 20.86) | 0.982 |  | IL-1α, $\mu$g/mL  median (IQR) | 0.72  (0.17 – 0.90) | 0.25  (0.23 – 1.99) | 0.231 |
| IL-1β, pg/mL  median (IQR) |  | 4.88  (2.84 – 6.70) | 5.52  (1.40 – 8.20) | 0.738 |  | IL-1β, $\mu$g/mL  median (IQR) | 0.16  (0.01 – 1.26) | 0.42  (0.06 – 0.63) | 0.622 |
| IL-1 ra, pg/mL  median (IQR) |  | 397.64  (320.44 – 538.12) | 454.76  (319.50 – 533.96) | 0.959 |  | IL-1 ra, $\mu$g/mL  median (IQR) | 205.65  (27.20 – 452.46) | 55.32  (23.04 – 79.17) | 0.214 |
| IL-2, pg/mL  median (IQR) |  | - | - | - |  | IL-2, $\mu$g/mL  median (IQR) | 0.28  (0.07 – 0.43) | 0.11  (0.03 – 0.15) | 0.222 |
| IL-3, pg/mL  median (IQR) |  | - | - | - |  | IL-3, $\mu$g/mL  median (IQR) | 0.74  (0.18 – 1.18) | 0.23  (0.07 – 1.00) | 0.546 |
| IL-4, pg/mL  median (IQR) |  | - | - | - |  | IL-4, $\mu$g/mL  median (IQR) | 0.05  (0.01 – 0.13) | 0.02  (0.01 – 0.03) | 0.489 |
| IL-5, pg/mL  median (IQR) |  | - | - | - |  | IL-5, $\mu$g/mL  median (IQR) | 0.10  (0.03 – 0.14) | 0.07  (0.03 – 0.12) | 0.529 |
| IL-6, pg/mL  median (IQR) |  | 11.50  (3.78 – 15.16) | 9.40  (2.64 – 27.64) | 0.618 |  | IL-6, $\mu$g/mL  median (IQR) | 0.78  (0.33 – 2.32) | 0.46  (0.21 – 1.11) | 0.340 |
| IL-7, pg/mL  median (IQR) |  | 12.40  (9.08 – 17.82) | 15.44  (11.72 – 17.60) | 1.000 |  | IL-7, $\mu$g/mL  median (IQR) | 0.23  (0.06 – 0.34) | 0.09  (0.04 – 0.14) | 0.168 |
| IL-8, pg/mL  median (IQR) |  | 8.76  (5.28 – 10.98) | 10.34  (8.54 – 17.90) | 0.342 |  | IL-8, $\mu$g/mL  median (IQR) | 16.98  (5.58 – 93.93) | 23.70  (8.94 – 77.27) | 0.796 |
| IL-10, pg/mL  median (IQR) |  | 28.52  (26.54 – 37.70) | 28.52  (12.46 – 59.72) | 0.447 |  | IL-10, $\mu$g/mL  median (IQR) | 2.91  (0.65 – 6.62) | 1.04  (0.54 – 1.37) | 0.297 |
| IL-12 p70, pg/mL  median (IQR) |  | - | - | - |  | IL-12p70, $\mu$g/mL  median (IQR) | 0.23  (0.03 – 0.37) | 0.07  (0.03 – 0.10) | 0.387 |
| IL-13, pg/mL  median (IQR) |  | - | - | - |  | IL-13, $\mu$g/mL  median (IQR) | 1.04  (0.33 – 1.70) | 0.38  (0.07 – 0.44) | 0.161 |
| IL-15, pg/mL  median (IQR) |  | 1.88  (1.28 – 3.00) | 1.56  (0.90 – 2.56) | 0.520 |  | IL-15, $\mu$g/mL  median (IQR) | 0.16  (0.10 – 0.17) | 0.05  (0.04 – 0.35) | 0.141 |
| IL-17, pg/mL  median (IQR) |  | - | - | - |  | IL-17, $\mu$g/mL  median (IQR) | 0.10  (0.02 – 0.14) | 0.04  (0.02 – 0.04) | 0.231 |
| IL-25, pg/mL  median (IQR) |  | - | - | - |  | IL-25, $\mu$g/mL  median (IQR) | 0.70  (0.07 – 1.01) | 0.13  (0.06 – 0.19) | 0.297 |
| IL-33, pg/mL  median (IQR) |  | 5.18  (4.42 – 7.78) | 6.12  (4.44 – 9.00) | 0.405 |  | IL-33, $\mu$g/mL  median (IQR) | 0.96  (0.72 – 2.33) | 0.69  (0.36 – 0.94) | 0.074 |
| ECP, kU/L  median (IQR) |  | 6.23  (4.00 – 11.10) | 6.20  (2.64 – 12.54) | 0.798 |  | ECP, g/L  median (IQR) | 0.12  (0.03 – 0.39) | 0.05  (0.01 – 0.42) | 0.546 |
| MPO, ng/mL  median (IQR) |  | 152.11  (130.50 – 221.81) | 279.11  (179.91 – 530.39) | 0.195 |  | MPO, mg/mL  median (IQR) | 0.06  (0.01 – 0.11) | 0.02  (0.01 – 0.04) | 0.168 |
| Total IgE, kU/L  median (IQR) |  | 174.50  (89.10 – 259.75) | 112.00  (71.38 – 282.25) | 0.505 |  | Total IgE, kU/L  median (IQR) | 90.72  (27.59 – 154.75) | 64.74  (22.90 – 121.37) | 0.730 |
| Periostin, ng/mL  median (IQR) |  | 245.94  (233.31 – 265.61) | 242.36  (215.43 – 270.04) | 0.878 |  | Periostin, mg/mL  median (IQR) | 5.36  (1.07 – 9.57) | 2.22  (0.96 – 3.66) | 0.436 |
| CD40L, pg/mL  median (IQR) |  | 164.96  (164.96 – 919.54) | 244.40  (164.96 – 4727.80) | 0.608 |  | CD40L, $\mu$g/mL  median (IQR) | 70.24  (12.99 – 93.43) | 20.60  (6.06 – 27.72) | 0.258 |
| EGF, pg/mL  median (IQR) |  | 24.70  (3.04 – 77.94) | 64.38  (7.28 – 284.48) | 0.320 |  | EGF, $\mu$g/mL  median (IQR) | 7.15  (1.70 – 17.39) | 2.33  (1.62 – 5.94) | 0.258 |
| Eotaxin, pg/mL  median (IQR) |  | 129.66  (98.68 – 175.42) | 123.46  (90.68 – 207.26) | 0.819 |  | Eotaxin, $\mu$g/mL  median (IQR) | 3.67  (0.69 – 5.42) | 1.28  (0.67 – 1.98) | 0.436 |
| FGF-basic, pg/mL  median (IQR) |  | 2.04  (0.64 – 3.42) | 3.42  (1.34 – 12.02) | 0.569 |  | FGF-basic, $\mu$g/mL  median (IQR) | 0.80  (0.25 – 1.11) | 0.23  (0.07 – 0.33) | 0.077 |
| Flt-3 Ligand, pg/mL  median (IQR) |  | 85.60  (64.68 – 107.20) | 89.12  (76.88 – 103.32) | 0.777 |  | Flt-3 Ligand, $\mu$g/mL  median (IQR) | 1.84  (0.49 – 3.52) | 1.08  (0.63 – 3.48) | 0.297 |
| G-CSF, pg/mL  median (IQR) |  | 71.72  (43.04 – 93.06) | 67.38  (43.12 – 92.68) | 0.878 |  | G-CSF, $\mu$g/mL  median (IQR) | 1.70  (0.75 – 46.92) | 5.80  (1.32 – 8.74) | 0.730 |
| GM-CSF, pg/mL  median (IQR) |  | 22.48  (16.12 – 25.84) | 19.94  (16.12 – 25.00) | 0.598 |  | GM-CSF, $\mu$g/mL  median (IQR) | 1.23  (0.37 – 3.95) | 0.57  (0.34 – 0.86) | 0.258 |
| Granzyme B, pg/mL  median (IQR) |  | - | - | - |  | Granzyme B, $\mu$g/mL  median (IQR) | 1.59  (0.36 – 6.25) | 0.89  (0.28 – 1.45) | 0.387 |
| CXCL-1, pg/mL  median (IQR) |  | 113.60  (67.96 – 178.90) | 93.96  (56.36 – 124.92) | 0.396 |  | CXCL-1, $\mu$g/mL  median (IQR) | 133.52  (27.45 – 376.42) | 60.28  (43.91 – 148.12) | 0.489 |
| CXCL-2, pg/mL  median (IQR) |  | 514.86  (409.42 – 914.56) | 883.04  (214.68 – 1310.2) | 0.959 |  | CXCL-2, $\mu$g/mL  median (IQR) | 3.49  (1.00 – 10.92) | 2.39  (1.80 – 5.65) | 0.605 |
| IFN-α, pg/mL  median (IQR) |  | - | - | - |  | IFN-α, $\mu$g/mL  median (IQR) | 0.48  (0.08 – 0.56) | 0.12  (0.04 – 0.13) | 0.190 |
| IFN-γ, pg/mL  median (IQR) |  | - | - | - |  | IFN-γ, $\mu$g/mL  median (IQR) | 0.65  (0.12 – 0.89) | 0.24  (0.09 – 0.28) | 0.340 |
| IP-10, pg/mL  median (IQR) |  | 119.60  (67.96 – 178.90) | 93.96  (56.36 – 124.92) | 0.396 |  | IP-10, $\mu$g/mL  median (IQR) | 2.69  (1.15 – 46.88) | 2.22  (1.36 – 4.89) | 0.436 |
| CCL-2, pg/mL  median (IQR) |  | 241.36  (192.62 – 305.40) | 310.32  (213.44 – 358.38) | 0.328 |  | CCL-2, $\mu$g/mL  median (IQR) | 2.31  (0.66 – 2.98) | 2.09  (0.96 – 2.50) | 0.796 |
| CCL-3, pg/mL  median (IQR) |  | 90.02  (78.12 – 120.74) | 95.06  (77.30 – 356.18) | 0.983 |  | CCL-3, $\mu$g/mL  median (IQR) | 0.93  (0.21 – 1.54) | 0.43  (0.16 – 1.53) | 0.863 |
| CCL-4, pg/mL  median (IQR) |  | 920.64  (706.54 – 1238.34) | 966.22  (889.56 –2333.78) | 0.505 |  | CCL-4, $\mu$g/mL  median (IQR) | 3.77  (0.29 – 10.42 | 4.07  (1.14 – 5.02) | 0.777 |
| CCL-5, ng/mL  median (IQR) |  | 34.60  (28.04 – 56.38) | 48.56  (22.84 – 67.04) | 0.721 |  | CCL-5, $m$g/mL  median (IQR) | 2.45  (1.07 – 7.88) | 0.81  (0.44 – 1.98) | 0.222 |
| CCL-19, pg/mL  median (IQR) |  | 89.98  (70.46 – 133.22) | 79.30  (59.46 – 118.18) | 0.798 |  | CCL-19, $\mu$g/mL  median (IQR) | 2.21  (0.66 – 9.29) | 1.79  (1.34 – 3.61) | 0.863 |
| CCL-20, pg/mL  median (IQR) |  | 8.50  (2.22 – 16.54) | 12.80  (8.52 – 18.32) | 0.204 |  | CCL-20, $\mu$g/mL  median (IQR) | 0.52  (0.18 – 3.65) | 0.38  (0.14 – 0.71) | 0.863 |
| PD-L1, pg/mL  median (IQR) |  | 39.46  (24.58 – 89.66) | 24.86  (15.78 – 29.46) | 0.150 |  | PD-L1, $\mu$g/mL  median (IQR) | 3.74  (0.75 – 5.15) | 0.96  (0.62 – 1.56) | 0.222 |
| PDGF-AA, pg/mL  median (IQR) |  | - | - | - |  | PDGF-AA, $\mu$g/mL  median (IQR) | 3.50  (1.58 – 4.87) | 1.73  (0.78 – 4.23) | 0.340 |
| PDGF-AB, pg/mL  median (IQR) |  | 2960.70  (1745.24 – 4031.50) | 3011.94  (1755.02 – 4039.84) | 0.959 |  | PDGF-AB, $\mu$g/mL  median (IQR) | 0.10  (0.02 – 0.24) | 0.05  (0.02 – 0.07) | 0.297 |
| TGF-α, pg/mL  median (IQR) |  | 9.64  (2.74 – 14.22) | 8.50  (4.66 – 11.16) | 0.939 |  | TGF-α, $\mu$g/mL  median (IQR) | 1.16  (0.35 – 1.65) | 0.33  (0.13 – 0.57) | 0.190 |
| TNF-α, pg/mL  median (IQR) |  | 3.24  (1.12 – 4.04) | 4.80  (1.72 – 7.12) | 0.136 |  | TNF-α, $\mu$g/mL  median (IQR) | 0.22  (0.05 – 0.95) | 0.17  (0.09 – 0.42) | 0.931 |
| TRAIL, pg/mL  median (IQR) |  | 37.12  (20.52 – 47.96) | 31.72  (20.24 – 53.92) | 0.939 |  | TRAIL, $\mu$g/mL  median (IQR) | 17.28  (4.10 – 44.67) | 5.73  (3.97 – 8.26) | 0.136 |
| VEGF, pg/mL  median (IQR) |  | 125.74  (98.12 – 156.32) | 113.22  (76.60 – 170.72) | 0.741 |  | VEGF, $\mu$g/mL  median (IQR) | 5.02  (1.62 – 7.85) | 2.11  (1.59 – 3.51) | 0.190 |

**Table S6.** The difference of biomarkers based on the facial pain score (FPS) improvement.

| Characteristics |  | Serum (N = 16) | | |  | Characteristics | Nasal secretion (N = 18) | | |
| --- | --- | --- | --- | --- | --- | --- | --- | --- | --- |
|  |  | Yes | No | *P* value |  |  | Yes | No | *P* value |
| IL-1α, pg/mL  median (IQR) |  | 15.52  (8.34 – 17.80) | 15.52  (12.62 – 16.68) | 0.978 |  | IL-1α, $\mu$g/mL  median (IQR) | 0.37  (0.07 – 0.97) | 0.25  (0.03 – 0.72) | 0.520 |
| IL-1β, pg/mL  median (IQR) |  | 3.18  (2.74 – 6.14) | 5.72  (4.66 – 7.38) | 0.392 |  | IL-1β, $\mu$g/mL  median (IQR) | 0.24  (0.02 – 0.42) | 0.14  (0.02 – 1.23) | 0.810 |
| IL-1 ra, pg/mL  median (IQR) |  | 376.08  (304.34 – 464.08) | 424.84  (355.36 – 501.82) | 0.299 |  | IL-1 ra, $\mu$g/mL  median (IQR) | 115.12  (41.53 – 256.50) | 44.32  (13.02 – 340.58) | 0.340 |
| IL-2, pg/mL  median (IQR) |  | - | - | - |  | IL-2, $\mu$g/mL  median (IQR) | 0.14  (0.04 – 0.39) | 0.12  (0.02 – 0.44) | 0.791 |
| IL-3, pg/mL  median (IQR) |  | - | - | - |  | IL-3, $\mu$g/mL  median (IQR) | 0.33  (0.09 – 1.24) | 0.25  (0.03 – 1.12) | 0.596 |
| IL-4, pg/mL  median (IQR) |  | - | - | - |  | IL-4, $\mu$g/mL  median (IQR) | 0.02  (0.00 – 0.16) | 0.02  (0.01 – 0.05) | 0.659 |
| IL-5, pg/mL  median (IQR) |  | - | - | - |  | IL-5, $\mu$g/mL  median (IQR) | 0.05  (0.02 – 0.17) | 0.08  (0.03 – 0.12) | 0.772 |
| IL-6, pg/mL  median (IQR) |  | 13.62  (2.64 – 15.68) | 13.62  (8.30 – 18.18) | 0.690 |  | IL-6, $\mu$g/mL  median (IQR) | 0.78  (0.53 – 1.57) | 0.46  (0.04 – 3.06) | 0.328 |
| IL-7, pg/mL  median (IQR) |  | 11.56  (7.12 – 14.26) | 15.44  (11.04 – 17.86) | 0.210 |  | IL-7, $\mu$g/mL  median (IQR) | 0.12  (0.05 – 0.27) | 0.09  (0.03 – 0.31) | 0.742 |
| IL-8, pg/mL  median (IQR) |  | 8.08  (3.92 – 9.74) | 10.34  (7.04 – 16.00) | 0.108 |  | IL-8, $\mu$g/mL  median (IQR) | 16.98  (8.57 – 52.82) | 21.45  (2.60 – 110.41) | 0.860 |
| IL-10, pg/mL  median (IQR) |  | 33.78  (28.52 – 41.62) | 28.52  (23.20 – 39.02) | 0.385 |  | IL-10, $\mu$g/mL  median (IQR) | 1.24  (0.58 – 5.36) | 1.24  (0.27 – 7.37) | 0.930 |
| IL-12 p70, pg/mL  median (IQR) |  | - | - | - |  | IL-12p70, $\mu$g/mL  median (IQR) | 0.10  (0.03 – 0.24) | 0.07  (0.01 – 0.42) | 1.000 |
| IL-13, pg/mL  median (IQR) |  | - | - | - |  | IL-13, $\mu$g/mL  median (IQR) | 0.61  (0.08 – 1.87) | 0.43  (0.05 – 1.04) | 0.479 |
| IL-15, pg/mL  median (IQR) |  | 1.78  (1.12 – 2.22) | 1.78  (1.66 – 2.88) | 0.419 |  | IL-15, $\mu$g/mL  median (IQR) | 0.15  (0.07 – 0.16) | 0.17  (0.04 – 0.35) | 0.705 |
| IL-17, pg/mL  median (IQR) |  | - | - | - |  | IL-17, $\mu$g/mL  median (IQR) | 0.41  (0.03 – 0.17) | 0.04  (0.01 – 0.10) | 0.520 |
| IL-25, pg/mL  median (IQR) |  | - | - | - |  | IL-25, $\mu$g/mL  median (IQR) | 0.14  (0.06 – 1.15) | 0.18  (0.05 – 0.70) | 0.596 |
| IL-33, pg/mL  median (IQR) |  | 4.44  (4.24 – 7.78) | 6.12  (4.34 – 8.60) | 0.519 |  | IL-33, $\mu$g/mL  median (IQR) | 0.85  (0.11 – 1.57) | 0.79  (0.46 – 2.37) | 0.791 |
| ECP, kU/L  median (IQR) |  | 5.77  (3.27 – 7.11) | 8.00  (4.27 – 13.79) | 0.351 |  | ECP, g/L  median (IQR) | 0.12  (0.02 – 0.23) | 0.11  (0.01 – 0.55) | 0.860 |
| MPO, ng/mL  median (IQR) |  | 178.13  (132.48 – 266.24) | 299.23  (134.92 – 481.17) | 0.606 |  | MPO, mg/mL  median (IQR) | 0.03  (0.02 – 0.10) | 0.02  (0.01 – 0.07) | 0.387 |
| Total IgE, kU/L  median (IQR) |  | 168.00  (63.20 – 330.00) | 120.00  (77.70 – 190.00) | 0.536 |  | Total IgE, kU/L  median (IQR) | 93.56  (36.74 – 173.06) | 64.74  (22.39 – 136.44) | 0.536 |
| Periostin, ng/mL  median (IQR) |  | 230.80  (173.57 – 271.13) | 256.82  (233.75 – 267.66) | 0.351 |  | Periostin, mg/mL  median (IQR) | 4.07  (1.18 – 10.19) | 2.22  (0.70 – 8.59) | 0.375 |
| CD40L, pg/mL  median (IQR) |  | 164.96  (164.96 –164.96) | 244.40  (164.96 – 2699.96) | 0.145 |  | CD40L, $\mu$g/mL  median (IQR) | 30.51  (8.12 – 81.87) | 21.99  (2.87 – 101.96) | 0.536 |
| EGF, pg/mL  median (IQR) |  | 2.04  (3.04 – 30.12) | 91.82  (26.08 – 257.24) | **0.039*** |  | EGF, $\mu$g/mL  median (IQR) | 7.15  (2.33 – 10.66) | 2.43  (0.97 – 8.81) | 0.375 |
| Eotaxin, pg/mL  median (IQR) |  | 125.88  (92.74 – 162.98) | 133.42  (96.00 – 200.34) | 0.778 |  | Eotaxin, $\mu$g/mL  median (IQR) | 1.84  (0.78 – 0.19) | 1.28  (0.22 – 4.31) | 0.536 |
| FGF-basic, pg/mL  median (IQR) |  | 3.42  (0.64 – 3.42) | 3.42  (0.64 – 10.38) | 0.088 |  | FGF-basic, $\mu$g/mL  median (IQR) | 0.40  (0.09 – 0.93) | 0.26  (0.10 – 0.99) | 0.791 |
| Flt-3 Ligand, pg/mL  median (IQR) |  | 85.86  (60.72 – 104.80) | 92.38  (75.24 – 104.78) | 0.778 |  | Flt-3 Ligand, $\mu$g/mL  median (IQR) | 1.25  (0.42 – 2.14) | 0.85  (0.13 – 3.48) | 0.659 |
| G-CSF, pg/mL  median (IQR) |  | 45.14  (40.44 – 98.52) | 68.00  (61.58 – 89.32) | 0.759 |  | G-CSF, $\mu$g/mL  median (IQR) | 3.98  (1.70 – 12.74) | 1.63  (0.97 – 8.66) | 0.375 |
| GM-CSF, pg/mL  median (IQR) |  | 20.80  (17.40 – 24.16) | 22.48  (13.98 – 25.84) | 0.864 |  | GM-CSF, $\mu$g/mL  median (IQR) | 0.76  (0.39 – 2.92) | 0.65  (0.16 – 2.36) | 0.724 |
| Granzyme B, pg/mL  median (IQR) |  | - | - | - |  | Granzyme B, $\mu$g/mL  median (IQR) | 1.59  (0.89 – 3.53) | 0.51  (0.21 – 1.58) | 0.211 |
| CXCL-1, pg/mL  median (IQR) |  | 100.74  (59.98 – 153.08) | 104.96  (66.62 – 143.40) | 0.980 |  | CXCL-1, $\mu$g/mL  median (IQR) | 126.90  (45.96 – 220.14) | 60.28  (17.08 – 237.05) | 0.536 |
| CXCL-2, pg/mL  median (IQR) |  | 571.34  (225.66 – 981.02) | 515.46  (457.88 – 956.00) | 0.918 |  | CXCL-2, $\mu$g/mL  median (IQR) | 3.49  (2.00 – 6.51) | 2.39  (0.65 – 8.68) | 0.479 |
| IFN-α, pg/mL  median (IQR) |  | - | - | - |  | IFN-α, $\mu$g/mL  median (IQR) | 0.15  (0.04 – 0.53) | 0.13  (0.03 – 0.48) | 0.596 |
| IFN-γ, pg/mL  median (IQR) |  | - | - | - |  | IFN-γ, $\mu$g/mL  median (IQR) | 0.31  (0.22 – 0.68) | 0.24  (0.02 – 1.07) | 0.479 |
| IP-10, pg/mL  median (IQR) |  | 100.74  (59.98 – 153.08) | 104.96  (66.62 – 143.40) | 0.980 |  | IP-10, $\mu$g/mL  median (IQR) | 2.69  (1.39 – 15.04) | 2.38  (0.91 – 5.81) | 0.724 |
| CCL-2, pg/mL  median (IQR) |  | 235.96  (183.02 – 379.06) | 251.86  (224.32 – 318.62) | 0.918 |  | CCL-2, $\mu$g/mL  median (IQR) | 2.31  (!.54 – 2.66) | 2.09  (0.33 – 2.50) | 0.596 |
| CCL-3, pg/mL  median (IQR) |  | 85.98  (75.50 – 125.46) | 10.1.00  (85.92 – 321.62) | 0.286 |  | CCL-3, $\mu$g/mL  median (IQR) | 0.46  (0.31 – 1.38) | 0.43  (0.06 – 1.62) | 0.930 |
| CCL-4, pg/mL  median (IQR) |  | 998.40  (841.06 – 1276.72) | 934.04  (813.44 – 1895.52) | 0.758 |  | CCL-4, $\mu$g/mL  median (IQR) | 4.07  (1.72 – 8.78) | 2.74  (0.46 – 4.92) | 0.810 |
| CCL-5, ng/mL  median (IQR) |  | 29.96  (21.38 – 54.32) | 34.78  (31.22 – 42.18) | 0.408 |  | CCL-5, $m$g/mL  median (IQR) | 1.88  (0.52 – 9.11) | 1.89  (0.26 – 2.45) | 0.659 |
| CCL-19, pg/mL  median (IQR) |  | 66.40  (57.78 – 146.70) | 92.18  (77.16 – 115.68) | 0.408 |  | CCL-19, $\mu$g/mL  median (IQR) | 5.67  (1.47 – 11.75) | 1.62  (0.43 – 2.45) | 0.069 |
| CCL-20, pg/mL  median (IQR) |  | 6.60  (3.90 – 18.98) | 12.20  (9.14 – 16.84) | 0.365 |  | CCL-20, $\mu$g/mL  median (IQR) | 0.52  (0.38 – 0.62) | 0.24  (0.09 – 1.72) | 0.362 |
| PD-L1, pg/mL  median (IQR) |  | 24.30  (12.94 – 41.44) | 33.48  (24.30 – 246.84) | 0.390 |  | PD-L1, $\mu$g/mL  median (IQR) | 1.45  (0.71 – 4.97) | 1.10  (0.20 – 5.33) | 0.791 |
| PDGF-AA, pg/mL  median (IQR) |  | - | - | - |  | PDGF-AA, $\mu$g/mL  median (IQR) | 3.48  (1.18 – 4.66) | 2.56  (0.78 – 5.38) | 1.000 |
| PDGF-AB, pg/mL  median (IQR) |  | 1881.76  (1565.6 – 5629.32) | 3179.94  (2710.88 – 39999.06) | 0.408 |  | PDGF-AB, $\mu$g/mL  median (IQR) | 0.09  (0.02 – 0.11) | 0.06  (0.01 – 0.30) | 0.791 |
| TGF-$\alpha$, pg/mL  median (IQR) |  | 3.90  (2.36 – 10.02) | 11.54  (7.34 – 13.84) | 0.179 |  | TGF-α, $\mu$g/mL  median (IQR) | 0.64  (0.13 – 1.55) | 0.48  (0.11 – 1.31) | 0.596 |
| TNF-$\alpha$,, pg/mL  median (IQR) |  | 4.02  (0.94 – 4.80) | 4.04  (2.10 – 5.96) | 0.479 |  | TNF-α, $\mu$g/mL  median (IQR) | 0.28  (0.10 – 0.53) | 0.17  (0.02 – 1.07) | 0.659 |
| TRAIL, pg/mL  median (IQR) |  | 33.96  (16.42 – 55.54) | 36.02  (21.88 – 49.04) | 1.000 |  | TRAIL, $\mu$g/mL  median (IQR) | 10.63  (6.24 – 30.79) | 5.73  (1.77 – 33.10) | 0.285 |
| VEGF, pg/mL  median (IQR) |  | 96.74  (67.34 – 117.94) | 133.56  (108.22 – 175.88) | 0.059 |  | VEGF, $\mu$g/mL  median (IQR) | 4.31  (1.98 – 6.17) | 2.11  (1.14 – 6.25) | 0.596 |

*, *P* < 0.05.

**Table S7.** The difference of biomarkers based on the nasal polyp score (NPS) improvement more than 2 points.

| Characteristics |  | Serum (N = 16) | | |  | Characteristics | Nasal secretion (N = 18) | | |
| --- | --- | --- | --- | --- | --- | --- | --- | --- | --- |
|  |  | Yes | No | *P* value |  |  | Yes | No | *P* value |
| IL-1α, pg/mL  median (IQR) |  | 7.28  (4.72 – 9.21) | 7.76  (6.31 – 8.68) | 0.700 |  | IL-1α, $\mu$g/mL  median (IQR) | 0.10  (0.03 – 1.18) | 0.76  (0.33 – 0.93) | **0.043*** |
| IL-1β, pg/mL  median (IQR) |  | 2.76  (1.03 – 3.07) | 2.86  (1.59 – 3.89) | 0.484 |  | IL-1β, $\mu$g/mL  median (IQR) | 0.24  (0.02 – 0.51) | 0.20  (0.04– 1.93) | 0.556 |
| IL-1 ra, pg/mL  median (IQR) |  | 185.22  (158.33 – 208.18) | 232.04  (206.80 – 281.40) | 0.071 |  | IL-1 ra, $\mu$g/mL  median (IQR) | 48.423  (18.56 – 88.16) | 231.073  (39.05 – 488.20) | 0.088 |
| IL-2, pg/mL  median (IQR) |  | - | - | - |  | IL-2, $\mu$g/mL  median (IQR) | 0.11  (0.02 – 0.15) | 0.33  (0.13 – 0.44) | 0.083 |
| IL-3, pg/mL  median (IQR) |  | - | - | - |  | IL-3, $\mu$g/mL  median (IQR) | 0.22  (0.05 – 0.33) | 1.08  (0.37 – 1.30) | 0.054 |
| IL-4, pg/mL  median (IQR) |  | - | - | - |  | IL-4, $\mu$g/mL  median (IQR) | 0.01  (0.00 – 0.04) | 0.04  (0.01 – 0.14) | 0.146 |
| IL-5, pg/mL  median (IQR) |  | - | - | - |  | IL-5, $\mu$g/mL  median (IQR) | 0.04  (0.02 – 0.11) | 0.10  (0.07 – 0.15) | 0.140 |
| IL-6, pg/mL  median (IQR) |  | 6.81  (4.15 – 7.83) | 4.81  (1.32 – 7.84) | 0.768 |  | IL-6, $\mu$g/mL  median (IQR) | 0.50  (0.12 – 1.01) | 0.92  (0.32 – 2.69) | 0.360 |
| IL-7, pg/mL  median (IQR) |  | 7.127  (5.65 – 8.64) | 5.606  (4.24 – 9.06) | 0.470 |  | IL-7, $\mu$g/mL  median (IQR) | 0.08  (0.03 – 0.12) | 0.25  (0.10 – 0.34) | 0.052 |
| IL-8, pg/mL  median (IQR) |  | 5.865  (3.52 – 6.20) | 4.041  (2.59 – 7.05) | 0.555 |  | IL-8, $\mu$g/mL  median (IQR) | 18.88  (2.60 – 65.05) | 30.96  (10.26 – 123.46) | 0.460 |
| IL-10, pg/mL  median (IQR) |  | 16.891  (11.60 – 21.46) | 14.258  (14.26 – 19.51) | 0.732 |  | IL-10, $\mu$g/mL  median (IQR) | 1.05  (0.43 – 1.31) | 4.13  (1.16 – 6.99) | 0.083 |
| IL-12 p70, pg/mL  median (IQR) |  | - | - | - |  | IL-12p70, $\mu$g/mL  median (IQR) | 0.06  (0.02 – 0.15) | 0.17  (0.08 – 0.40) | 0.122 |
| IL-13, pg/mL  median (IQR) |  | - | - | - |  | IL-13, $\mu$g/mL  median (IQR) | 0.35  (0.05 – 0.63) | 1.17  (0.39 – 1.79) | 0.122 |
| IL-15, pg/mL  median (IQR) |  | 0.887  (0.78 – 1.30) | 0.887  (0.56 – 1.55) | 0.897 |  | IL-15, $\mu$g/mL  median (IQR) | 0.07  (0.04 – 0.35) | 0.17  (0.12 – 0.17) | 0.179 |
| IL-17, pg/mL  median (IQR) |  | - | - | - |  | IL-17, $\mu$g/mL  median (IQR) | 0.03  (0.01 – 0.06) | 0.10  (0.04 – 0.15) | 0.11 |
| IL-25, pg/mL  median (IQR) |  | - | - | - |  | IL-25, $\mu$g/mL  median (IQR) | 0.11  (0.06 – 0.31) | 0.71  (0.10 – 1.08) | 0.122 |
| IL-33, pg/mL  median (IQR) |  | 3.06  (2.12 – 3.89) | 3.06  (2.12 – 4.70) | 0.697 |  | IL-33, $\mu$g/mL  median (IQR) | 0.64  (0.37 – 0.86) | 1.26  (0.43 – 2.47) | 0.083 |
| ECP, kU/L  median (IQR) |  | 5.28  (2.847 – 7.56) | 10.12  (3.61 – 13.34) | 0.252 |  | ECP, g/L  median (IQR) | 0.04  (0.02 – 0.16) | 0.24  (0.05 – 0.55) | 0.237 |
| MPO, ng/mL  median (IQR) |  | 178.13  (134.92 – 279.11) | 229.23  (132.48 – 591.83) | 0.351 |  | MPO, mg/mL  median (IQR) | 0.02  (0.01 – 0.03) | 0.08  (0.02 – 0.11) | **0.046*** |
| Total IgE, kU/L  median (IQR) |  | 139.00  (112.00 – 264.50) | 77.80  (69.30 – 280.00) | 0.408 |  | Total IgE, kU/L  median (IQR) | 37.40  (16.77 – 70.15) | 142.82  (96.52 – 185.68) | **0.004**** |
| Periostin, ng/mL  median (IQR) |  | 262.42  (231.94 – 271.65) | 230. 80  (214.83– 245.21) | 0.091 |  | Periostin, mg/mL  median (IQR) | 1.61  (0.68 – 2.89) | 6.97  (2.47 – 9.88) | 0.068 |
| CD40L, pg/mL  median (IQR) |  | 82.48  (82.48 – 1059.34) | 82.48  (82.48 – 585.531) | 0.427 |  | CD40L, $\mu$g/mL  median (IQR) | 19.87  (3.72 – 26.57) | 76.05  (22.73 – 97.70) | 0.101 |
| EGF, pg/mL  median (IQR) |  | 10.02  (1.52 – 91.23) | 16.44  (1.52 – 45.91) | 0.979 |  | EGF, $\mu$g/mL  median (IQR) | 2.38  (1.35 – 7.56) | 6.60  (2.51 – 20.76) | 0.203 |
| Eotaxin, pg/mL  median (IQR) |  | 81.49  (58.24 – 117.13) | 62.94  (42.67 – 66.71) | 0.291 |  | Eotaxin, $\mu$g/mL  median (IQR) | 1.17  (0.30 – 1.81) | 3.99  (1.68 – 5.53) | **0.037*** |
| FGF-basic, pg/mL  median (IQR) |  | 1.71  (0.32 – 2.16) | 1.71  (0.32 – 6.83) | 0.933 |  | FGF-basic, $\mu$g/mL  median (IQR) | 0.22  (0.08 – 0.40) | 0.87  (0.28 – 1.17) | 0.122 |
| Flt-3 Ligand, pg/mL  median (IQR) |  | 42.93  (39.67 – 50.37) | 44.56  (28.70 – 55.36) | 0.737 |  | Flt-3 Ligand, $\mu$g/mL  median (IQR) | 0.74  (0.22 – 1.12) | 1.99  (0.78 – 3.95) | 0.083 |
| G-CSF, pg/mL  median (IQR) |  | 33.16  (20.92 – 45.16) | 34.00  (21.17 – 48.10) | 0.681 |  | G-CSF, $\mu$g/mL  median (IQR) | 1.422  (0.872 – 8.702) | 7.106  (2.128 – 64.011) | 0.146 |
| GM-CSF, pg/mL  median (IQR) |  | 11.24  (8.27 – 12.92) | 8.70  (7.85 – 12.92) | 0.594 |  | GM-CSF, $\mu$g/mL  median (IQR) | 0.52  (0.25 – 0.76) | 2.08  (0.66 – 4.27) | **0.027*** |
| Granzyme B, pg/mL  median (IQR) |  | - | - | - |  | Granzyme B, $\mu$g/mL  median (IQR) | 0.70  (0.23 – 1.27) | 1.97  (0.62 – 7.62) | 0.101 |
| CXCL-1, pg/mL  median (IQR) |  | 50.37  (18.46 – 71.16) | 45.93  (43.59 – 74.16) | 0.738 |  | CXCL-1, $\mu$g/mL  median (IQR) | 47.77  (33.97 – 91.89) | 194.74  (78.59 – 433.02) | **0.027*** |
| CXCL-2, pg/mL  median (IQR) |  | 262.84  (155.83 – 466.01) | 257.732  (252.01 – 514.48) | 0.681 |  | CXCL-2, $\mu$g/mL  median (IQR) | 2.19  (1.18 – 3.80) | 6.64  (2.24 – 11.20) | 0.068 |
| IFN-α, pg/mL  median (IQR) |  | - | - | - |  | IFN-α, $\mu$g/mL  median (IQR) | 0.11  (0.03 – 0.16) | 0.50  (0.13 – 0.57) | 0.068 |
| IFN-γ, pg/mL  median (IQR) |  | - | - | - |  | IFN-γ, $\mu$g/mL  median (IQR) | 0.23  (0.05 – 0.27) | 0.66  (0.20 – 0.98) | 0.173 |
| IP-10, pg/mL  median (IQR) |  | 50.37  (18.46 – 71.16) | 45.93  (43.58 – 74.16) | 0.738 |  | IP-10, $\mu$g/mL  median (IQR) | 1.72  (1.23 – 3.65) | 10.43  (2.34 – 50.94) | **0.034*** |
| CCL-2, pg/mL  median (IQR) |  | 156.23  (97.41 – 175.56) | 124.06  (117.98 – 142.09) | 0.837 |  | CCL-2, $\mu$g/mL  median (IQR) | 1.32  (0.70 – 2.42) | 2.32  (1.97 – 3.22) | 0.315 |
| CCL-3, pg/mL  median (IQR) |  | 50.50  (39.56 – 140.10) | 44.55  (37.75 – 53.31) | 0.487 |  | CCL-3, $\mu$g/mL  median (IQR) | 0.46  (0.31 – 1.38) | 0.43  (0.06 – 1.62) | 0.930 |
| CCL-4, pg/mL  median (IQR) |  | 499.20  (430.50 – 1395.06) | 457.71  (346.71 – 561.61) | 0.408 |  | CCL-4, $\mu$g/mL  median (IQR) | 3.25  (0.53 – 4.34) | 4.87  (0.26 – 11.23) | 0.573 |
| CCL-5, ng/mL  median (IQR) |  | 24.01  (12.76 – 31.40) | 15.92  (13.70 – 28.74) | 0.758 |  | CCL-5, $m$g/mL  median (IQR) | 0.69  (0.33 – 3.08) | 2.33  (1.64 – 8.39) | 0.146 |
| CCL-19, pg/mL  median (IQR) |  | 46.09  (37.82 – 57.84) | 32.28  (28.34 – 73.35) | 0.351 |  | CCL-19, $\mu$g/mL  median (IQR) | 1.54  (0.77 – 2.92) | 4.06  (1.77 – 9.90) | 0.122 |
| CCL-20, pg/mL  median (IQR) |  | 5.20  (1.95 – 7.13) | 6.10  (3.30 – 9.28) | 0.626 |  | CCL-20, $\mu$g/mL  median (IQR) | 0.38  (0.17 – 0.61) | 0.57  (0.16 – 5.09) | 0.446 |
| PD-L1, pg/mL  median (IQR) |  | 14.73  (12.151 – 34.35) | 12.70  (6.47 – 20.72) | 0.778 |  | PD-L1, $\mu$g/mL  median (IQR) | 0.97  (0.45 – 1.52) | 4.35  (1.14 – 5.24) | 0.101 |
| PDGF-AA, pg/mL  median (IQR) |  | - | - | - |  | PDGF-AA, $\mu$g/mL  median (IQR) | 1.84  (0.73 – 3.28) | 4.08  (2.03 – 5.34) | 0.122 |
| PDGF-AB, pg/mL  median (IQR) |  | 1437.50  (774.78 – 1991.16) | 1507.66  (940.88 – 2031.98) | 0.687 |  | PDGF-AB, $\mu$g/mL  median (IQR) | 0.04  (0.02 – 0.08) | 0.11  (0.05 – 0.28) | 0.054 |
| TGF-α, pg/mL  median (IQR) |  | 4.25  (1.95 – 6.16) | 4.25  (1.18 – 7.30) | 0.980 |  | TGF-α, $\mu$g/mL  median (IQR) | 0.38  (0.12 – 0.60) | 1.23  (0.42 – 1.70) | 0.101 |
| TNF-α, pg/mL  median (IQR) |  | 2.397  (1.24 – 3.56) | 1.24  (0.47 – 2.02) | 0.056 |  | TNF-α, $\mu$g/mL  median (IQR) | 0.16  (0.07 – 0.35) | 0.24  (0.15 – 1.16) | 0.274 |
| TRAIL, pg/mL  median (IQR) |  | 16.38  (8.76 – 26.15) | 18.01  (12.58 – 24.52) | 0.857 |  | TRAIL, $\mu$g/mL  median (IQR) | 6.33  (3.10 – 8.95) | 24.03  (4.73 – 50.46) | 0.237 |
| VEGF, pg/mL  median (IQR) |  | 67.52  (44.84 – 88.68) | 52.19  (48.37 – 58.97) | 0.241 |  | VEGF, $\mu$g/mL  median (IQR) | 2.09  (1.18 – 3.28) | 5.25  (2.66 – 8.17) | 0.068 |

*, *P* < 0.05.

**TABLE S8.** Diagnostic accuracy measures of optimal cutoff values of the biomarkers of serum or nasal secretion for predicting clinical response.

| Biomarker | AUC | 95% Confidence interval | *P* value | Cutoff | Sensitivity | Specificity | Youden index |
| --- | --- | --- | --- | --- | --- | --- | --- |
| SNOT-22 improvement > 8.9 points | | | | | | | |
| Serum CCL-3 | 0.836 | 0.586–1.000 | 0.036 | 42.175 | 90.0% | 80.0% | 0.700 |
| Serum CCL-4 | 0.909 | 0.740–1.000 | 0.011 | 443.381 | 90.0% | 80.0% | 0.700 |
| NCS non-response | | | | | | | |
| Serum IL-8 | 0.883 | 0.711-1.000 | 0.013 | 5.020 | 83.3% | 90% | 0.733 |
| FPS non-response | | | | | | | |
| Serum EGF | 0.810 | 0.586-1.000 | 0.039 | 15.750 | 77.8% | 85.7% | 0.635 |
| NPS none and less response | | | | | | | |
| NS eotaxin | 0.788 | 0.550-1.000 | 0.041 | 1.410 | 87.5% | 70.0% | 0.575 |
| NS GM-CSF | 0.813 | 0.594-1.000 | 0.026 | 1.086 | 62.5% | 90% | 0.525 |
| NS CXCL-1 | 0.812 | 0.596-1.000 | 0.026 | 130.207 | 75.0% | 90.0% | 0.650 |
| NS IP-10 | 0.800 | 0.570-1.000 | 0.033 | 5.530 | 62.5% | 100.0% | 0.625 |
| NS IL-1α  | 0.788 | 0.550-1.000 | 0.041 | 0.237 | 87.5% | 70.0% | 0.575 |
| NS MPO | 0.781 | 0.537–1.000 | 0.046 | 0.039 | 75.0% | 90.0% | 0.650 |
| NS IgE | 0.900 | 0.730–1.000 | 0.004 | 88.555 | 87.50% | 90.00% | 0.775 |
